# Identification of the antibacterial action mechanism of diterpenoids through transcriptome profiling

**DOI:** 10.3389/fmicb.2022.945023

**Published:** 2022-07-26

**Authors:** Keumok Moon, Sungmin Hwang, Hyeon-Jeong Lee, Eunhye Jo, Jeong Nam Kim, Jaeho Cha

**Affiliations:** ^1^Microbiological Resource Research Institute, Pusan National University, Busan, South Korea; ^2^Clean Energy Research Center, Korea Institute of Science and Technology, Seoul, South Korea; ^3^Department of Integrated Biological Science, College of Natural Sciences, Pusan National University, Busan, South Korea; ^4^Department of Microbiology, College of Natural Sciences, Pusan National University, Busan, South Korea

**Keywords:** *Aralia continentalis*, diterpenoids, antibacterial activity, *Streptococcus mutans*, transcriptome analysis

## Abstract

Effective antibacterial substances of *Aralia continentalis* have anti-biofilm and bactericidal activity to the oral pathogen *Streptococcus mutans*. In this study, three compounds extracted from *A. continentalis* were identified as acanthoic acid, continentalic acid, and kaurenoic acid by NMR and were further investigated how these diterpenoids affect the physiology of the *S. mutans*. When *S. mutans* was exposed to individual or mixed fraction of diterpenoids, severe growth defects and unique morphology were observed. The proportion of unsaturated fatty acids in the cell membrane was increased compared to that of saturated fatty acids in the presence of diterpenoids. Genome-wide gene expression profiles with RNA-seq were compared to reveal the mode of action of diterpenoids. *Streptococcus mutans* commonly enhanced the expression of 176 genes in the presence of the individual diterpenoids, whereas the expression of 232 genes was considerably reduced. The diterpenoid treatment modulated the expression of genes or operon(s) involved in cell membrane synthesis, cell division, and carbohydrate metabolism of *S. mutans*. Collectively, these findings provide novel insights into the antibacterial effect of diterpenoids to control *S. mutans* infection, which causes human dental caries.

## Introduction

The second-largest microbial consortia, composed of over 700 different bacterial species, are present in the oral cavity after the intestines in the human body (Aas et al., 2005). Some of these bacteria are associated with oral disease. Periodontitis is a common inflammatory disease that is initiated from forming complex multi-layers of microorganisms such as *Streptococcus*, *Actinomyces*, *Veillonella*, *Porphyromonas*, and *Fusobacterium* (Jenkinson and Lamont, 2005). Streptococci, especially, *S. mutans*, are attributed to tooth decay or dental caries. Surprising, *S. mutans* is constituted a minor proportion (<1% of the active oral microbiome) on healthy tooth surfaces, even in caries lesions (Simón-Soro et al., 2014); however, its unique metabolic properties of biofilm formation and diverse carbohydrate catalysis promote the cariogenic processes and finally result in dental cavities (Lemos et al., 2019). Since decayed teeth are irreversible and cannot be regenerated, the most effective way to prevent dental caries and periodontal disease is to remove plaque.

Several approaches have been used to maintain oral hygiene and protect the tooth from the bacterial infection. Since discovering antibiotics, they have been widely used for human therapy including the oral cavity (Alaki et al., 2009); however, the increasing ineffectiveness and the emerging multi-drug resistance strains have been taken into consideration (Davies and Davies, 2010). Other compounds (i.e., sodium fluoride) were also used to prevent tooth from bacterial plaque forming; nevertheless, these chemicals are toxic to oral mucosal (Jeng et al., 1998). Natural products which derived from plants have been raised as alternative antimicrobial agents to improve oral health in that the relatively easy acquisition and low cost (Lee et al., 2019).

*Aralia continentalis* is a perennial herb species belonging to the family Araliaceae. It has been used for its anti-inflammatory, anti-diabetic, anti-cancer, and antimicrobial activity in therapeutics (Lim et al., 2009; Jung et al., 2012). Among the natural compounds from *A. continentalis*, the major secondary metabolites, continentalic acid (CA) and kaurenoic acid (KA), exhibit an antibacterial activity against several bacteria, such as *S. mutans*, *Staphylococcus aureus*, *Enterococcus* strains, and *Porphyromonas gingivalis* (Wilkens et al., 2002; Jeong et al., 2006, 2008, 2010, 2013; Martins et al., 2018). CA exerts considerable inhibitory effects on the ability of *S. mutans* to form biofilms (Jeong et al., 2010). This is because the terpenoid interferes the formation of cell membrane by intercalating into the phospholipid bilayer (Urzúa et al., 2008). Wilkens et al. reported that KA exhibits bacteriolytic activity against Gram-positive, *Bacillus cereus* (Wilkens et al., 2002). In addition, KA not only inhibits the growth, acid production, and biofilm formation of *S. mutans* at a concentration of 4 μg/ml but reduces the expression of genes involved in virulence factors from *S. mutans* (Jeong et al., 2013).

Though many studies have identified the chemical structure of diterpenoids from the extracts from plants and demonstrated the effects as an antibacterial agent focusing on bacterial morphology, physiological characteristics, and expression levels of key genes (Jeong et al., 2010, 2013), the underlying mechanism for the antibacterial activity of plant secondary metabolites is still remained to be discovered. In this report, three diterpenoids, such as CA, KA, and acanthoic acid (AA), were isolated from *A. continentalis* and characterized their antibacterial effects on *S. mutans*. Furthermore, genome-wide gene expression profiles by RNA-seq were investigated in the presence of the diterpenoids to understand the antibacterial mechanism. This study will be valuable in designing new dental hygiene regimens and drugs to prevent dental caries by *S. mutans* infection.

## Materials and methods

### Chemicals and reagents

The roots of *A. continentalis* were purchased in a local market in Seoul, Korea, in May 2020. All solvents for extraction were of low particulate grade and purchased from SK chemicals (Ulsan, Korea). Methanol and acetonitrile were purchased from Burdick & Jackson (Ulsan, Korea). Brain Heart Infusion (BHI) broth was purchased from Difco (Sparks, MD). All other reagents were purchased from Sigma-Aldrich (St. Louis, MO).

### Preparation of *Aralia continentalis* extracts and purification of antibacterial compounds

The roots were ground with a food processor and sterilized at 121°C for 15 min. Fifty grams of the roots was extracted with 1 l of ethanol by sonication at 50°C for 60 min, then the supernatant was collected. The process was repeated three times, and the collected supernatants were mixed and filtered (No. 2, Toyo Roshi Kaisha, Ltd., Japan). The supernatant was concentrated with a rotary vacuum evaporator (Eyela, Japan) under reduced pressure at 50°C. The ethanol extract (6.49 g) was suspended in 10% ethanol and fractionated with *n*-hexane (1 g). The hexane fraction was concentrated through rotary vacuum evaporation at 50°C and then stored at −20°C until use. The hexane fraction was loaded on a silica gel column (6 × 40 cm) equilibrated with chloroform/ethyl acetate (19:1, v/v) and partitioned. Using a preparative recycling high-performance liquid chromatography (HPLC) system (LC-918, JAI, Japan) coupled to an ultraviolet (UV) detector, the fraction of the diterpenoid mixture (DM, 460 mg) was further isolated. Approximately 3 ml of the reaction mixture was loaded on an octadecylsilyl (ODS)-A column (5 μm particle size, 2 × 25 cm; YMC Co., Kyoto, Japan). The column was equilibrated with methanol/acetonitrile/0.1% trifluoroacetic acid (42.75:52.25:5, v/v/v) and then split into three fractions (Supplementary Figure 1).

### Thin-layer chromatography and HPLC analyses of antibacterial compounds

A TLC silica gel plate (Merck KGaA, Darmstadt, Germany) was used to identify the antibacterial compounds. First, TLC plates were developed in a developing solution containing 5% formic acid in methanol/*n*-hexane (1:5, v/v). Next, they were observed after dipping them in a solution containing 1% *N*-(1-naphtyl)-ethylenediamine and 20% sulfuric acid in methanol, followed by heating at 110°C for 5 min.

HPLC analysis was conducted using a Nanospace instrument (Shiseido, Tokyo, Japan). Identification of antibacterial compounds was performed by reverse-phase HPLC using an ODS-A (4.6 × 250 mm, YMC Co.) column. A gradient elution was used to achieve chromatographic separation with mobile phases A (water containing 0.1% trifluoroacetic acid) and B (acetonitrile/methanol = 55:45, v/v). The mobile phase was 95% B. The flow rate was 0.7 ml/min, and the column was maintained at 30°C. The injection volume was 5 μl and the analytical run time for each sample was 60 min. Optical density (O.D.) was measured at 205 nm.

### Nuclear magnetic resonance analysis

The ^1^H and ^13^C NMR spectra of antibacterial compounds were obtained using an Avance 600 MHz NMR spectrometer (Bruker, Germany). The sample was dissolved in dimethyl sulfoxide-*d_6_* at 24°C with tetramethylsilane as a chemical shift reference.

### Mass spectrometry analysis

The antibacterial compounds were analyzed *via* a mass spectrometry system. The mass spectrometer (maXis HD, Bruker, Billerica, MA) was operated in positive mode with a capillary voltage of 4,500 V and an end-plate offset of −500 V. The scanning mass range (m/z) was from 50 to 1,600. Nitrogen was used as the nebulizing gas at a flow rate of 2 l/min, a temperature of 200°C, and a pressure of 2.0 bar. The possible elemental compositions of each peak were determined by the SmartFormula program using a mass accuracy of <2 ppm error.

### Antibacterial activity assay

*Streptococcus mutans* UA159 was grown in BHI broth at 37°C in a 5% CO_2_ incubator. The microtiter broth dilution method determined the minimum inhibitory concentration (MIC) of antibacterial compounds (Wiegand et al., 2008). In brief, DM, AA, CA, or KA was prepared by serial twofold dilutions at a concentration of 1–64 μg/ml in BHI broth. *Streptococcus mutans* was added at 5 × 10^5^ CFU/ml to each sterile medium in 96 well plates. The plates were incubated at 37°C in a 5% CO_2_ incubator for 24 h. Growth inhibition was monitored by measuring the absorbance at 600 nm (Xiang et al., 2017). *Streptococcus mutans* UA159 was grown up to the mid-exponential phase (O.D._600_ = 0.4–0.5) to confirm the effect of diterpenoids during the exponential growth phase and then treated with diterpenoids at a concentration of 4 or 40 μg/ml.

### Biofilm assay

Biofilm formation was measured according to the previous method with modifications (Xiang et al., 2019). Briefly, *S. mutans* UA159 was cultured into BHI medium, grown until reaching the O.D._600_ = 0.4–0.5, and 0.2% sucrose was added into the medium. The bacterial cultures were then aliquoted in a 96-well plate, 4 μg/ml of each diterpenoid was added to BHI broth, and incubated at 37°C in a 5% CO_2_ incubator for 24 h. The culture medium of each well was decanted, and attached biofilms were stained with 0.1% crystal violet. The bound dye was extracted with 95% ethanol and measured spectrophotometrically at 570 nm.

### Scanning electron microscopy

An SEM (Gemini500, Carl Zeiss Co., Ltd., Germany) was used to observe the morphological changes in cells after treatment with diterpenoids. *Streptococcus mutans* UA159 was grown up to the mid-exponential phase (O.D._600_ = 0.4–0.5) and then treated with DM at the concentration of 4 or 40 μg/ml. Bacterial cells treated with diterpenoids were harvested and washed three times with phosphate-buffered saline (PBS). The bacterial cells were fixed in 4% glutaraldehyde for 2 h and washed five times with PBS. The cells were washed for 15 min each with increasing ethanol concentration (30%, 50%, 70%, 80%, 90%, 95%, and 100%).

### Fatty acid composition analysis

To confirm the change in the fatty acid composition of *S. mutans* by diterpenoid treatment, *S. mutans* was grown to the mid-exponential phase (O.D._600_ = 0.4–0.5) and treated with DM at a concentration of 4 or 40 μg/ml. Total lipid extraction was conducted using the Bligh and Dyer method (Bligh and Dyer, 1959). Approximately 5 mg of lipid was extracted per sample (100 ml aliquot of culture). Each preparation was used to prepare membrane fatty acid esters by the addition of 0.2 ml of toluene and 0.4 ml of 1% H_2_SO_4_ in methanol. The mixture was heated for 30 min; samples were cooled and fatty acids were extracted *via* the addition of 1 ml of hexane and 1 ml of H_2_O. The hexane phase was then evaporated under nitrogen gas, and the fatty acid methyl esters were reconstituted in hexane. Gas chromatography was performed on a 7890GC/5975C MSD (Agilent) equipped with an SP2560 capillary column (100 m × 0.25 mm × 0.2 μm). The column was kept at 15 lb/in^2^ and 220°C, and nitrogen was used as the carrier gas. A Nu-Chek Prep standard no. 68A was used to determine retention times and the identity of fatty acids derived from *S. mutans*.

### RNA sequencing, data processing, and analysis

*Streptococcus mutans* UA159 was grown to the mid-exponential phase (O.D._600_ = 0.4–0.5) in BHI broth at 37°C in a 5% CO_2_ incubator. The antibacterial compounds of *A. continentalis* were added to the cells at a concentration of 4 μg/ml and the cells were further incubated for 1 h at 37°C in a 5% CO_2_ incubator. Each sample was prepared in biological triplicate. The cells were harvested, resuspended in 1 ml of RNAprotect Bacteria Reagent (Qiagen, Venlo, Netherlands), and maintained at room temperature for 10 min. The cells were resuspended in 50 mM Tris–HCl buffer containing 10 mM EDTA and 0.4% SDS and then mechanically disrupted using Bead Beater 16 (Biospec Products Inc., Bartlesville, OK). Total RNA was isolated using an RNeasy Mini Kit (Qiagen, Valencia, CA) and the RNA concentration was measured using a NanoDrop Spectrophotometer (NanoDrop™ 2000 Spectrophotometer, Thermo Fisher Scientific, Waltham, MA). Prior to the sequencing library construction, the quality and integrity of the extracted RNA were checked using a 2,100 Bioanalyzer (Agilent Technologies, Santa Clara, CA), and the quantity was measured by quantitative polymerase chain reaction (qPCR) as per the Illumina qPCR Quantification Protocol Guide. One microliter of total RNA was used for the construction of cDNA libraries. Ribosomal RNA was removed by Ribo-Zero™ Removal Kits (Bacteria) and then converted to cDNA using TruSeq RNA Library Prep Kit v2 (Illumina, San Diego, CA). Paired-end sequencing of the constructed cDNA libraries was performed by Illumina NovaSeq. Raw sequencing reads were assessed. In addition, further adapter, poor-quality, or short reads (<10 bp) were trimmed using FastQC v.0.11.7.^1^ Additionally, the preprocessed reads were aligned to the reference genome of *S. mutans* UA159^2^ using Bowtie2 (Langmead and Salzberg, 2012), followed by sorting and indexing by SAMtools (Li et al., 2009). The number of reads mapped to each transcript was counted by HTSeq (Anders et al., 2015). The read counts were normalized, and differentially expressed genes (DEGs) were identified using DESeq2 (Love et al., 2014). Genes with a log_2_ fold change >1 and a false discovery rate determined by Benjamini–Hochberg (BH) correction for multiple hypothesis testing of <0.05 were considered DEGs. Functional enrichment of DEGs was performed using the hypergeometric test from the eggNOG 4.5 orthology database (Huerta-Cepas et al., 2016). All RNA-seq data were deposited in the NCBI database under accession no. GSE202812.

### Statistical analysis

All experiments were performed in triplicate. Data were analyzed by employing the one-way analysis of variance test using the SigmaPlot software (version 12.5, Systat, San Jose, CA), and expressed as the mean ± standard deviation. Also, the mean value was considered to be significantly different at *p* < 0.05, *p* < 0.005, and *p* < 0.001.

## Results

### Isolation and identification of major antibacterial compounds in *Aralia continentalis* extracts

**Figure 1:**
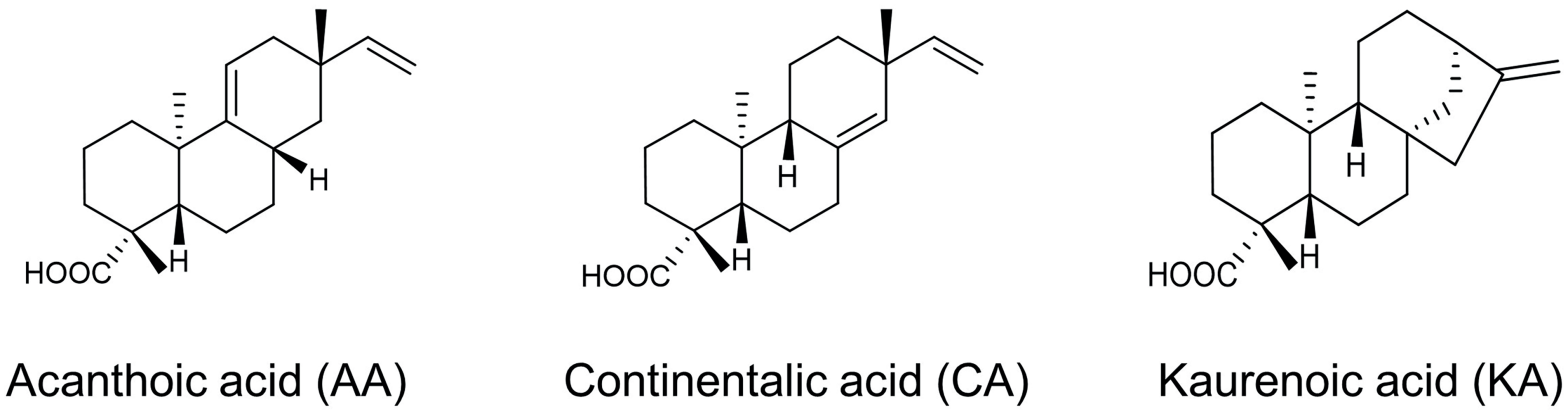
Antibacterial compounds derived from *Aralia continentalis.*

*Aralia continentalis*, a member of the Araliaceae family, produces secondary metabolites that contribute to cellular activities, such as anti-inflammatory and antimicrobial activity (Lim et al., 2009; Jung et al., 2012). First, we attempted to determine the chemical compounds possessing bioactive properties in *A. continentalis*. The active ingredients exhibiting high antibacterial activity were isolated from the roots using organic solvent extraction, silica gel chromatography, and preparative recycling HPLC (prepLC). In the prepLC, three peaks isolated were weighed as 71.4 mg, 141.4 mg, and 93.0 mg, respectively (Supplementary Figure 1A). By using HPLC to measure the purity of each compound, it was 91%, 90%, and 95%, while the yield of those chemicals was 0.14%, 0.28%, and 0.19%, respectively. The chemical structure of the three isolates was further analyzed using ^1^H-NMR and ^13^C-NMR and identified as AA, CA, and KA, respectively (Figure 1; Supplementary Table 1). The ^1^H-NMR and ^13^C-NMR data of the CA and AA were compared with the previous report (Lee et al., 2009). While both CA and KA were the major antimicrobial products, AA was the first compound that is isolated from the roots of *A. continentalis* (Wilkens et al., 2002; Jeong et al., 2006, 2008, 2010, 2013; Martins et al., 2018). AA, which is a pimarane-type diterpenoid, was initially isolated from the root bark of *Acanthopanax koreanum* and was reported to have antibacterial activity against oral and skin bacteria (Kim et al., 1988; Kim, 2006).

### Effects of diterpenoids on the phenotypes of *Streptococcus mutans* UA159

**Figure 2:**
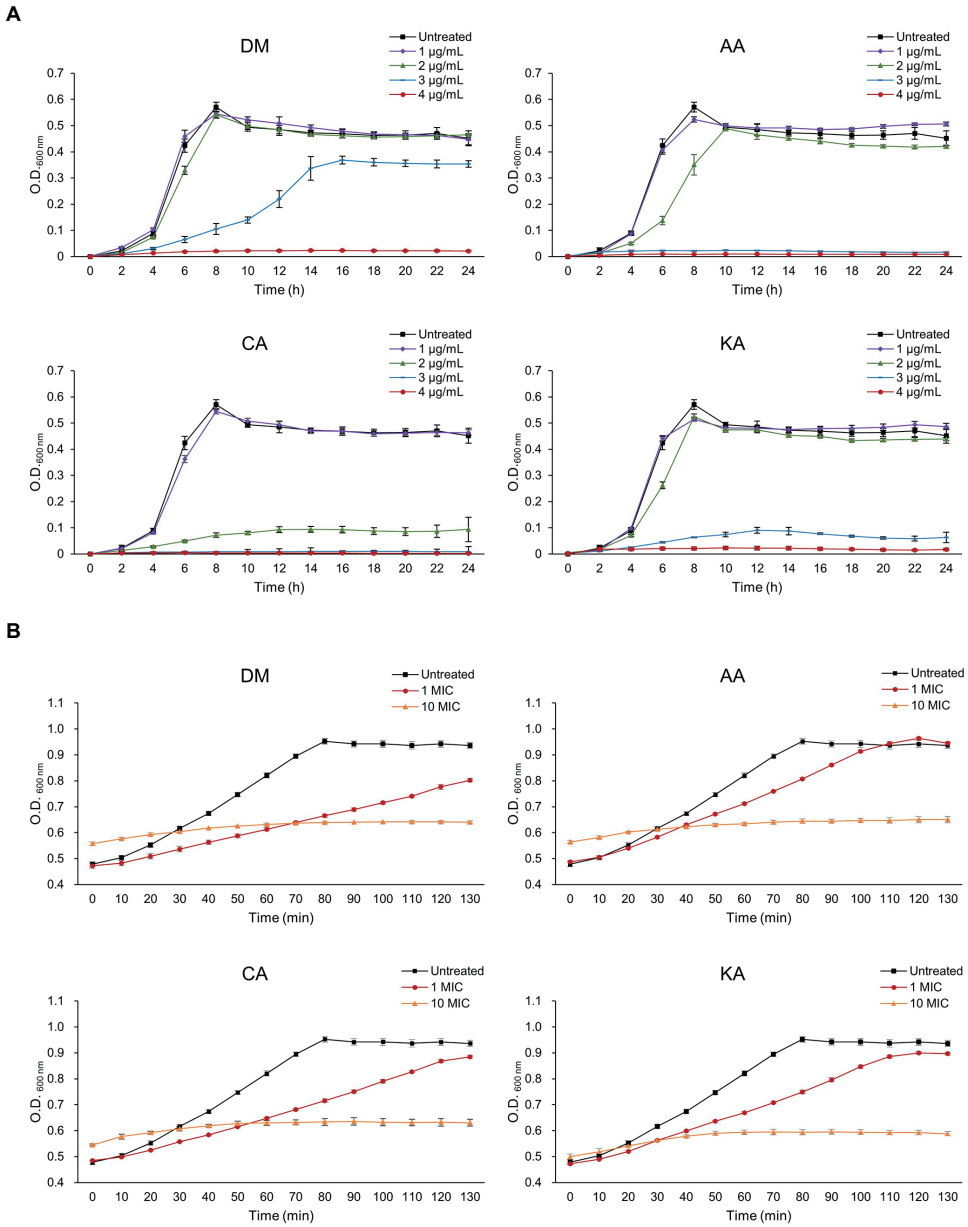
The effect of diterpenoids on the growth of *S. mutans*. Diterpenoids were added to the bacteria in **(A)** the lag phase and **(B)** the mid exponential phase.

Bacterial vulnerability toward antibiotics is shown to be concentration-dependent. Ampicillin and tetracycline can be useful antibiotics to treat the growth of *S. mutans* with the MIC <1 μg/ml (Järvinen et al., 1995). However, the advent of multi-drug resistance bacteria by overuse of antibiotics has threatened human healthcare and promoted a discovery of alternatives. To this end, we tested whether the natural diterpenoids affect the growth of *S. mutans*, an etiological microorganism of dental caries. Initially, bacterial growth was observed under the DM, AA, CA, and KA isolated from *A. continentalis* using the final concentrations of 1, 2, 3, and 4 μg/ml, respectively. As shown in Figure 2A, all three diterpenoids substantially inhibited the growth of *S. mutans* above 3 μg/ml and completely inhibited the growth at 4 μg/ml, which corresponded to the MIC. These results corresponded well with those of previous reports in that diterpenoids extracted from *A. continentalis* inhibited the growth of *S. mutans* (Jeong et al., 2010, 2013). The cells were grown to the mid-exponential phase and were further treated with 4 or 40 μg/ml diterpenoids. These steps were taken to understand the inhibitory mechanism of diterpenoids on *S. mutans*. Although the growth of *S. mutans* was inhibited by diterpenoids at 4 μg/ml, it recovered after ~2 h. However, the growth was completely inhibited by diterpenoids at a 40 μg/ml concentration corresponding to 10 MIC (Figure 2B).

**Figure 3:**
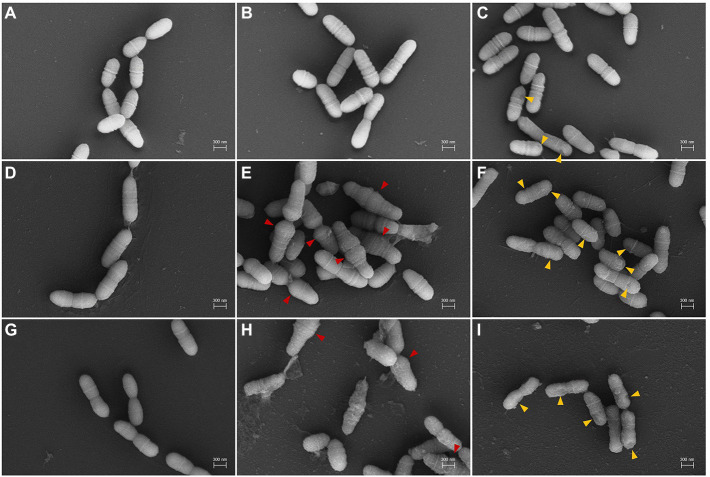
Scanning electron microscopy (SEM) micrographs of *S. mutans* treated with DM. **(A–C)**, 1 h treatment; **(D–F)**, 6 h treatment; **(G–I)**, 24 h treatment. **(A,D,G)**, untreated; **(B,E,H)**, treated with 4 μg/ml; **(C,F,I)**, treated with 40 μg/ml. Red and orange arrows indicate aberrant morphology and abnormal extracellular coating by DM treatment, respectively.

SEM analysis was performed to observe the morphological growth inhibition dependent on the diterpenoid concentration in the mid-stage of exponential growth (Figure 3). SEM analyses of *S. mutans* treated with 4 μg/ml of DM for 6 and 24 h showed significant alterations in the pathogen morphology (Figures 3E,H). In the untreated group, the bacterial cells grew normally with a complete appearance and smooth surfaces (Figures 3A,D,G). Cell morphology was maintained after treatment with 40 μg/ml of DM for 6 and 24 h. However, some extracellular coatings were missing (Figures 3F,I). Furthermore, bacteria treated with DM for 24 h demonstrated cell membrane damage, which occurred owing to cell debris surrounding the bacteria (Figures 3H,I).

*Streptococcus mutans* metabolizes carbohydrates to synthesize adhesive glucans, and then, the cells attach to the tooth surface to form a biofilm (Matsumoto-Nakano, 2018). Inhibition of biofilm formation is a very important activity because it inhibits the initial adhesion of *S. mutans* to the tooth surface. CA and KA are known to inhibit biofilm formation in *S. mutans* (Jeong et al., 2010, 2013). Biofilm formation was inhibited by 24 h exposure of both individual and mixed diterpenoids. In addition, genes involved in biofilm synthesis, such as *gtfB, gtfC*, and *gbpB*, were downregulated by the diterpenoids (Supplementary Figure 2; Supplementary Table 2).

Reportedly, *S. mutans* alters the proportion of membrane fatty acid composition as one of its adaptation mechanisms to environmental changes. Therefore, to investigate the effects of diterpenoids on the fatty acid composition of *S. mutans*, the cells were treated with DM (4 and 40 μg/ml) for 1 or 6 h (Table 1). After 1 h of treatment, the proportion of saturated fatty acids increased to 84.5 ± 0.8, 90.1 ± 1.1, and 93.1 ± 4.4 as the diterpenoid concentration increased to 0, 4, and 40 μg/ml. In contrast, the monounsaturated fatty acids gradually decreased to 15.5% ± 0.8, 9.8% ± 1.3, and 5.1% ± 3.7%. However, after 6 h of treatment, the proportion of saturated fatty acids decreased to 90.7% ± 1.4, 85.8% ± 3.0, and 87.0% ± 3.3%, and monounsaturated fatty acids increased to 9.3% ± 1.4, 13.9% ± 2.8, and 9.7% ± 1.7%. Moreover, the proportion of polyunsaturated fatty acids increased with an increase in time and concentration. At an acidic pH, *S. mutans* decreases proton permeability by increasing the proportion of unsaturated and long-chain fatty acids. Thus, fatty acid redistribution in the membrane is essential for low pH survival and contributes to *S. mutans* acid adaptation (Fozo and Quivey, 2004; Fozo et al., 2007). Therefore, changes in fatty acid composition of the membrane also accompany the adaptation of this bacterium to antibacterial substances.

**Table 1:** Fatty acid composition of *Streptococcus mutans* UA159.

	Control		DM_4 μg/ml		DM_40 μg/ml	
	1 h	6 h	1 h	6 h	1 h	6 h
C12:0	0.8 ± 0.1	0.7 ± 0.1	0.4 ± 0.1	0.5 ± 0.0	0.6 ± 0.2	0.6 ± 0.0
C14:0	4.0 ± 0.4	3.3 ± 0.6	2.1 ± 0.3	2.4 ± 0.2	2.1 ± 0.5	2.4 ± 0.2
C15:0	0.1 ± 0.0	0.1 ± 0.1	0.1 ± 0.1	0.1 ± 0.0	0.1 ± 0.0	0.2 ± 0.1
C16:0	54.1 ± 0.8	56.4 ± 1.3	55.5 ± 0.4	53.3 ± 1.8	56.2 ± 2.2	52.2 ± 2.9
C16:1	0.8 ± 0.1	0.5 ± 0.5	0.7 ± 0.2	0.7 ± 0.3	0.3 ± 0.5	0.7 ± 0.1
C17:0	0.3 ± 0.0	0.3 ± 0.0	0.3 ± 0.0	0.3 ± 0.0	0.4 ± 0.1	0.5 ± 0.0
C18:0	24.5 ± 1.1	29.2 ± 1.2	30.7 ± 1.3	27.9 ± 1.6	32.4 ± 3.4	28.3 ± 1.4
C18:1n9c	3.8 ± 0.1	2.7 ± 0.4	2.5 ± 0.3	2.8 ± 0.7	2.3 ± 0.7	3.1 ± 0.4
C18:2n6c	ND	ND	0.1 ± 0.2	0.3 ± 0.3	1.8 ± 0.8	3.3 ± 1.9
C20:0	0.6 ± 0.0	0.6 ± 0.1	0.7 ± 0.1	1.1 ± 0.2	0.6 ± 0.1	0.8 ± 0.1
C20:1	8.6 ± 0.3	3.7 ± 0.6	4.0 ± 0.6	8.4 ± 2.0	1.7 ± 1.5	3.3 ± 0.7
C21:0	ND	ND	ND	ND	ND	ND
C20:2	ND	ND	ND	ND	ND	0.4 ± 0.4
C22:0	0.1 ± 0.0	0.1 ± 0.1	0.2 ± 0.0	0.3 ± 0.0	0.4 ± 0.1	0.8 ± 0.3
C22:1n9	2.4 ± 0.7	2.3 ± 0.1	2.6 ± 0.6	1.9 ± 0.3	0.8 ± 1.4	2.5 ± 0.8
C23:0	ND	ND	ND	ND	ND	0.2 ± 0.1
C24:0	ND	ND	ND	ND	0.3 ± 0.2	0.7 ± 0.4
SFA^a^	84.5 ± 0.8	90.7 ± 1.4	90.1 ± 1.1	85.8 ± 3.0	93.1^**^ ± 4.4	87.0 ± 3.3
MSFA^b^	15.5 ± 0.8	9.3 ± 1.4	9.8 ± 1.3	13.9 ± 2.8	5.1^***^ ± 3.7	9.7 ± 1.7
PSFA^c^	ND	ND	0.1 ± 0.2	0.3 ± 0.3	1.8^*^^,^^#^ ± 0.8	3.3^*^^,^^#^ ± 1.9

Data are expressed as the average % fatty acid ± standard deviation (*n* = 3). ^Significant differences were observed when compared with the controls.^

* *p* < 0.05;

** *p* < 0.005;

*** *p* < 0.001.

Significant differences were observed when compared with DM_4μg/ml.

# *p* < 0.05.

a Saturated fatty acid.

b Monounsaturated fatty acid.

c Polyunsaturated fatty acid.

### Transcription profiling of *Streptococcus mutans* UA159 treated with diterpenoids

**Figure 4:**
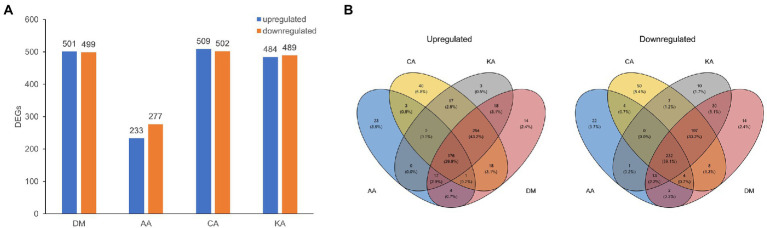
DEGs detected in comparison between diterpenoid-untreated and diterpenoid-treated samples. *Streptococcus mutans* was treated with DM, AA, CA, and KA. **(A)** Up- and downregulated number of DEGs, **(B)** Venn diagram showing unique and shared DEGs. DEGs with >1.0-fold change and *p* < 0.05 were clustered across all samples.

To examine the transcriptome, DM, AA, CA, or KA was separately treated with *S. mutans* for 1 h. After each diterpenoid treatment, RNA-seq was performed using the total mRNA isolated from cells in the exponential growth phase. DEGs were identified by comparing RNA sequence data from *S. mutans* treated with the diterpenoids. The heatmap and principal component analysis plot indicated that the overall expression trends in the cells treated with DM, AA, CA, and KA were similar to each other (Supplementary Figure 2). After applying the classification threshold (the absolute value of log_2_ fold change ≥1 and *p* ≤ 0.05), a total of 1,000, 500, 1,011, and 973 DEGs by DM, AA, CA, and KA were identified, respectively, in comparison with untreated cells (Figure 4A). In addition, volcano plots were generated to examine the upregulated or downregulated DEG patterns among the treatment conditions (Supplementary Figure 3C). The results revealed a similar number of upregulated and downregulated DEGs by DM, AA, CA, and KA (501, 223, 509, and 484 upregulated and 499, 277, 502, and 489 downregulated). The Venn diagram indicated that DM, CA, and KA share 447 upregulated genes and 436 downregulated genes, of which 176 upregulated genes and 232 downregulated genes are shared with AA (Figure 4B). This result suggests that CA, KA, and AA have similar antimicrobial mechanisms against *S. mutans*.

**Figure 5:**
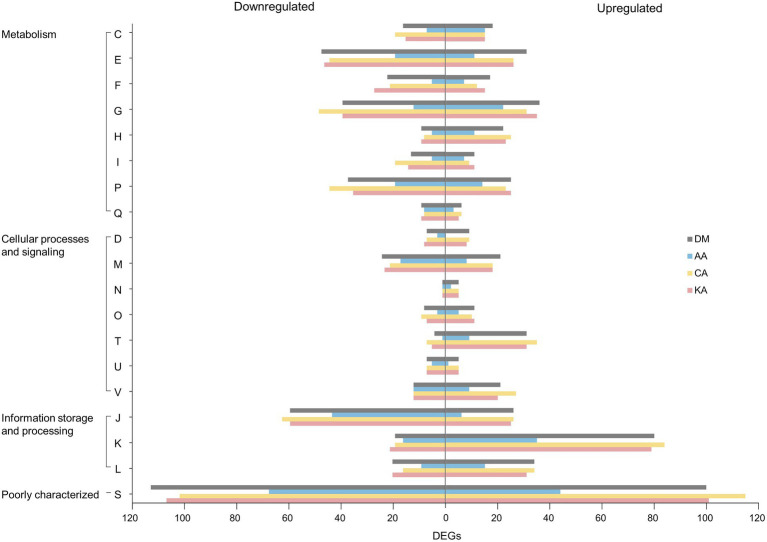
Cluster of Orthologous Groups (COG) classification of the identified DEGs. The alphabetical codes represent the following functional categories: C, energy production and conversion; E, amino acid transport and metabolism; F, nucleotide transport and metabolism; G, carbohydrate transport and metabolism; H, coenzyme metabolism; I, lipid metabolism; P, inorganic ion transport and metabolism; Q, secondary metabolite biosynthesis, transport, and catabolism; D, cell division and chromosome partitioning; M, cell envelope biogenesis and outer membrane; N, cell motility and secretion; O, post-translational modification, protein turnover, and chaperones; T, signal transduction mechanisms; U, intracellular trafficking, secretion, and vesicular transport; and V, defense mechanisms; J, translation, ribosomal structure, and biogenesis; K, transcription; L, DNA replication, recombination, and repair; S, function unknown (|log_2_| ≥ 1, *p*_adj_ < 0.05).

The COG database classified DEGs into three major categories: metabolism, cellular processes and signaling, and information storage and processing (Figure 5). Coenzyme-related genes showed a remarkable upregulation in response to all types of diterpenoids in the metabolism category (Supplementary Table 3). In contrast, genes associated with amino acid and secondary metabolites were downregulated (Supplementary Tables 4, 5). In the cellular processes and signaling category, higher amounts of downregulated genes were noted than upregulated genes. In the information storage and processing category, translation-related genes were the most downregulated among all categories. However, genes associated with transcription were the most upregulated in all categories. The metabolic features of *S. mutans* UA159 were further investigated by mapping the transcriptome of the strain treated with diterpenoids to the Kyoto Encyclopedia of Genes and Genomes pathways (Supplementary Figure 4). It showed that the metabolism of carbohydrates, amino acids, nucleotides, fatty acids, cofactors, and vitamins was highly expressed under normal conditions (Supplementary Figure 4A). However, the metabolism of carbohydrates, fatty acids, nucleotides, and amino acids was severely affected by diterpenoids (Supplementary Figure 4B).

### Transcriptome analysis of carbohydrate transport and metabolism

Bacteria uptake and metabolize different types of carbohydrates for their survival. The sugars in the cytoplasm are mainly utilized for energy production through the glycolytic pathway. They are also utilized in the biosynthesis pathway of various bacterial components, such as virulence factors, including biofilm and lipoteichoic acid, cell walls, nucleic acids, and amino acids (Lemos et al., 2019). *Streptococcus mutans* can metabolize a greater variety of carbohydrates than any other Gram-positive organism identified till date, which is one of the main survival strategies of this bacterium (Ajdić et al., 2002). *Streptococcus mutans* encodes 14 phosphoenolpyruvate-dependent phosphotransferase systems (PTSs) and two ATP-binding cassette (ABC) transporters. These are membrane proteins involved in the internalization of oligosaccharides. The transcriptome analysis of *S. mutans* UA159 showed that the major carbohydrate metabolism gene expression was highly diminished by diterpenoids (Table 2; Supplementary Figure 4). Genes involved in carbohydrate uptake, i.e., PTSs for maltose, sucrose, β-glucoside, lactose, cellobiose, and mannose were downregulated. In contrast, PTSs for mannitol, sorbitol, and trehalose were upregulated in the cells treated with diterpenoids. *msmE*, *gtfA*, and *dexB* were upregulated by AA, KA, and DM in *msm, malXFGK,* and *glg* operon for the metabolism of oligosaccharides. However, *aga, msmFGK,* and *malFG* were downregulated only by *CA.* In the *glg* operon, there was no tendency to either up- or downregulate the genes based on the diterpenoid type. With the exception of oligosaccharide metabolism in *S. mutans*, carbohydrate transport and metabolism of this strain by CA, KA, and DM were mostly lower than those of untreated bacteria. In this strain, the transport and metabolism of carbohydrates were also lower after AA treatment in comparison with the untreated bacteria, but were less affected than CA, KA, and DM.

**Table 2:** Differential gene expression associated with carbohydrate uptake and metabolism.

Locus ID	Annotation	Gene	Gene expression (log_2_ FC)^*^			
			AA	CA	KA	DM
SMU_RS09355	PTS transporter subunit EIIC	*ptsG*	−1.39	−3.48	−2.10	−2.37
SMU_RS08435	PTS beta-glucoside transporter subunit IIBCA	*scrA*	−0.68	−3.83	−2.06	−2.13
SMU_RS04505	PTS transporter subunit EIIC	*bglP*	−0.62	−4.11	−2.20	−2.14
SMU_RS06770	PTS transporter subunit EIIC	*lacE*	−0.65	−1.42	−1.45	−1.69
SMU_RS07235	PTS cellobiose transporter subunit IIC	*ptcC*	−1.32	−2.91	−2.83	−3.10
SMU_RS08590	PTS mannose transporter subunit IIAB	*ptnA*	−1.45	−3.44	−2.06	−2.42
SMU_RS08600	PTS mannose/fructose/sorbose transporter family subunit IID		−0.83	−2.76	−1.55	−1.79
SMU_RS08595	PTS mannose/fructose/sorbose transporter subunit IIC	*ptnC*	−1.08	−3.16	−1.87	−2.14
SMU_RS05445	PTS sugar transporter subunit IIA	*mtlA2*	3.10	1.12	2.38	2.39
SMU_RS05455	PTS mannitol transporter subunit IICBA	*mtlA1*	3.34		1.65	1.41
SMU_RS01555	PTS glucitol/sorbitol transporter subunit IIA		0.88	1.68	1.79	1.85
SMU_RS09325	PTS system trehalose-specific EIIBC component	*pttB*	2.94		2.56	2.43
SMU_RS04080	Extracellular solute-binding protein	*msmE*	1.23	0.86	2.31	2.26
SMU_RS04095	Sucrose phosphorylase	*gtfA*	0.98		1.69	1.71
SMU_RS04105	Alpha-glucosidase	*dexB*	1.28		1.23	1.25
SMU_RS04075	Alpha-galactosidase	*aga*		−1.21		
SMU_RS04085	Sugar ABC transporter permease	*msmF*		−1.76	−0.33	−0.45
SMU_RS04090	Carbohydrate ABC transporter permease	*msmG*		−1.20	0.26	0.14
SMU_RS04100	sn-glycerol-3-phosphate ABC transporter ATP-binding protein UgpC	*msmK*		−1.95	−0.66	−0.59
SMU_RS07115	Sugar ABC transporter permease	*malF*		−1.99	−0.69	−0.62
SMU_RS07120	Sugar ABC transporter permease	*malG*		−1.36	−0.11	0.03
SMU_RS02605	ROK family glucokinase	*glk*	−0.61	−3.08	−1.60	−1.62
SMU_RS01525	Glucose-6-phosphate isomerase	*pgi*		−1.43	−1.09	−1.23
SMU_RS05480	6-Phosphofructokinase	*pfkA*	−0.78	−1.77	−1.82	−1.80
SMU_RS00520	Fructose-bisphosphate aldolase	*fbaA*	−0.53	−1.81	−1.83	−1.89
SMU_RS03370	Triose-phosphate isomerase	*tpiA*	−0.48	−1.48	−1.39	−1.37
SMU_RS01775	Type I glyceraldehyde-3-phosphate dehydrogenase	*gapC*		−1.87	−1.71	−1.82
SMU_RS01445	Transketolase	*tkt*	−1.68	−3.84	−3.28	−3.36
SMU_RS05685	Phosphopentomutase	*deoB*	−0.83	−2.30	−2.24	−2.23
SMU_RS05690	Ribose-5-phosphate isomerase	*rpiA*	−0.80	−2.29	−2.36	−2.34
SMU_RS00440	Fructan beta-fructosidase	*fruA*		−2.97	−1.59	−1.68
SMU_RS03200	Phosphoenolpyruvate-protein phosphotransferase			−2.15	−1.44	−1.36
SMU_RS00590	1-Phosphofructokinase		1.01	2.16	2.91	2.69

* Gene expression is expressed using absolute log_2_ fold change (FC) values with adjusted *p*-values <0.05. Red and blue correspond to up- or downregulated gene expression levels, respectively.

Many genes associated with glycolysis, pentose phosphate metabolism, and fructose and mannose metabolism were downregulated 2-14-fold by diterpenoids (Table 2), including glucose kinase (*glk*), glucose-6-phosphate isomerase (*pgi*), 6-phosphofructokinase (*pfk*), fructose-1,6-biphosphate aldolase (*fbaA*), triosephosphate isomerase (*tpiA*), glyceraldehyde-3-phosphate dehydrogenase (*gapC*), transketolase (*tkt*), phosphopentomutase (*deoB*), ribose 5-phosphate isomerase A (*rpiA*), and fructan β-fructosidase (*fruA*).

### Transcriptome analysis of fatty acid metabolism

The genes involved in fatty acid synthesis encoding SMU.1734–1739, SMU.1741, and SMU.1744, including β-ketoacyl-ACP synthase II (*fabF*), ACP dehydratase (*fabZ*), were mostly downregulated by diterpenoid treatment. Interestingly, *trans*-2-*cis*-3-decenoyl-ACP isomerase (*fabM*), which encodes SMU.1746c involved in the unsaturated fatty acid synthesis, was upregulated twofold by AA (Supplementary Table 6). This finding is consistent with the increase in unsaturated fatty acids after AA treatment. These changes in the proportion of saturated and unsaturated fatty acids are known as one of the important adaptation mechanisms of *S. mutans* in response to external stimuli (Fozo and Quivey, 2004; Bajerski et al., 2017; Wang et al., 2018).

### Transcriptome analysis of cell division and cell envelope biogenesis

The cell wall of Gram-positive bacteria protects cells from environmental stress tolerance, antibiotic susceptibility, host immune evasion, and overall virulence (Kovacs et al., 2019). Several clusters of genes are involved in cell division present in the UA159 genome. In addition, there are more than 60 proteins responsible for cell envelope biogenesis (Ajdić et al., 2002). Among them, *ftsZ*, *ftsX,* and *ftsW* involved in cell division were downregulated, whereas *ftsL* was upregulated by DM, CA, and KA. *ezrA* and *mreCD,* which affect the cell shape, were also downregulated by diterpenoids (Table 3). The extracellular matrix of cariogenic biofilms is mainly composed of exopolysaccharides (EPS), lipoteichoic acids, and extracellular DNA (Klein et al., 2015). *gtfBC* and *ftf* involved in EPS synthesis were also downregulated in *S. mutans*. In the *dltXABCD* operon causing d-alanylation of teichoic acids, *dltXCD* genes were upregulated by CA, KA, and DM. Rhamnosyltransferase and glucosyltransferase were upregulated among the rhamnose-glucose polysaccharide (RGP)-related genes of *S. mutans*. These compounds function in a manner similar to teichoic acids in other Gram-positive bacteria. Meanwhile, in contrast, the ABC transporter was downregulated. *lrgAB* and *cidAB*, putative membrane proteins that share structural features with bacteriophage-encoded holin family proteins, were downregulated 2-32-fold by CA, KA, and DM. *Streptococcus mutans* exhibits antimicrobial peptide resistance using two-component systems (TCSs), which sense and adapt to the environment (Ajdić et al., 2002; Kawada-Matsuo and Komatsuzawa, 2017). Among them, *liaFSR*, *bceRS*, and *lcrRS,* which sense and regulate the cell envelope stress, were upregulated 2-12-fold by CA, KA, and DM. Altogether, these results imply that the genes involved in cell protection, communication with the environment, maintenance of cell shape, stability, and rigidity of cells are considerably affected by diterpenoids.

**Table 3:** Differential gene expression associated with cell division and cell envelope biogenesis.

Locus ID	Annotation	Gene	Gene expression (log_2_ FC)^*^			
			AA	CA	KA	DM
SMU_RS02645	Cell division protein	*ftsZ*	−0.65	−1.59	−1.56	−1.55
SMU_RS05890	FtsW/RodA/SpoVE family cell cycle protein	*ftsX*		−0.77	−0.91	−1.06
SMU_RS03360	FtsW/RodA/SpoVE family cell cycle protein	*ftsW*	−0.84	−1.66	−1.64	−1.57
SMU_RS02185	Cell division protein	*ftsL*		2.27	2.18	2.49
SMU_RS05875	Septation ring formation regulator	*ezrA*	−0.83	−1.58	−1.75	−1.62
SMU_RS00180	Rod shape-determining protein	*mreC*	−1.16	−1.29	−1.31	−1.27
SMU_RS00185	Rod shape-determining protein	*mreD*	−2.00	−5.23	−4.52	−3.61
SMU_RS04620	Glucosyltransferase-I	*gtfB*	−0.77	−2.41	−1.93	−1.81
SMU_RS04625	Glucosyltransferase-SI	*gtfC*	−1.11	−3.10	−2.81	−2.57
SMU_RS09270	Levansucrase	*ftf*	−0.97	−1.37	−0.54	−0.88
SMU_RS07685	Teichoic acid D-Ala incorporation-associated protein	*dltX*		6.25	6.45	5.70
SMU_RS07670	D-alanine--poly(phosphoribitol) ligase subunit	*dltC*	0.89	1.38	1.57	1.49
SMU_RS07665	D-alanyl-lipoteichoic acid biosynthesis protein	*dltD*	0.53	1.24	1.39	1.39
SMU_RS01255	Undecaprenyl/decaprenyl-phosphate alpha-N-acetylglucosaminyl 1-phosphate transferase	*rgpG*		−1.29	−1.36	−1.39
SMU_RS03815	dTDP-4-dehydrorhamnose reductase	*rmlD*		0.89	0.96	1.12
SMU_RS03830	ABC transporter permease	*rgpC*	−0.73	−1.28	−1.25	−1.27
SMU_RS03840	Glycosyltransferase	*rgpE*		2.12	2.07	2.22
SMU_RS03845	Alpha-L-Rha alpha-1,3-L-rhamnosyltransferase	*rgpF*		1.45	1.56	1.67
SMU_RS03855	Hypothetical protein	*rpgH*		1.23	1.55	1.59
SMU_RS03860	Glycosyltransferase family 2 protein	*rpgI*		0.72	1.24	1.34
SMU_RS02745	Antiholin-like protein	*lrgB*	−2.19	−4.59	−3.40	−3.87
SMU_RS02750	CidA/LrgA family protein	*lrgA*	−3.42	−5.09	−3.90	−4.59
SMU_RS07720	LrgB family protein		−1.03	−0.44	−1.50	−1.63
SMU_RS07725	CidA/LrgA family protein		−1.01		−1.34	−1.74
SMU_RS02340	Response regulator transcription factor	*liaR*	1.31	3.33	2.80	3.01
SMU_RS02335	Sensor histidine kinase	*liaS*	1.32	3.58	3.10	3.34
SMU_RS04640	Response regulator transcription factor	*bceR*	1.50	2.46	2.05	2.37
SMU_RS04645	Sensor histidine kinase	*bceS*		3.28	2.91	3.19
SMU_RS05280	Response regulator transcription factor	*lcrR*		2.49	2.12	2.41
SMU_RS05275	HAMP domain-containing histidine kinase	*lcrS*		2.61	2.30	2.64

* Gene expression is expressed using absolute log_2_ fold change (FC) values with adjusted *p*-values < 0.05. Red and blue correspond to up- or downregulated gene expression levels, respectively.

### Transcriptome analysis of amino acid transport and metabolism

*Streptococcus mutans* possesses all amino acid biosynthetic pathways. Thus, they can grow in any minimal medium free of amino acids if thiosulfate is provided for cysteine biosynthesis (Ajdić et al., 2002). Cysteine is an essential component for the growth of *S. mutans* and is associated with coenzyme A biosynthesis (Carlsson, 1970; Terleckyj and Shockman, 1975; Spry et al., 2008). Diterpenoid treatment resulted in the expression of genes involved in synthesizing amino acids, such as serine, threonine, lysine, glutamate, histidine, arginine, and proline and branched-chain amino acids, such as valine, isoleucine, and leucine, to decrease (Supplementary Figure 4). In contrast, the expression of genes for cysteine, methionine, and aromatic amino acid synthesis was increased. The genes encoding the synthesis of amino acid permease, polar amino acid, aspartate, glutamate, and glutamine permease were downregulated. Furthermore, the cysteine transport system permease was upregulated. The high expression of genes involved in cysteine and methionine synthesis was in concert with the upregulation of genes for coenzyme A transport and metabolism (Supplementary Table 7).

### Secondary metabolite biosynthesis, transport, and catabolism

*Streptococcus mutans* possesses various secondary metabolites. These include bacteriocins (mutacins) and one hybrid polyketide/non-ribosomal peptide type compound. They have an important role in interspecies or inter-kingdom interactions in dental biofilms (Ajdić et al., 2002; Xie et al., 2017). However, most genes encoding the biosynthesis of secondary metabolites were downregulated, particularly the mutanobactin operon-related genes (SMU 1338c–1347c). They were downregulated 2-14-fold by all types of diterpenoids (Supplementary Table 8). Mutanobactin, which is a hybrid polyketide/non-ribosomal peptide type compound, has an important role in increasing bacterial hydrophobicity, promoting bacterial adhesion and biofilm formation, and regulating a range of stress tolerance, including oxygen and hydrogen peroxide resistance in *S. mutans* (Wu et al., 2010; Li et al., 2020). Therefore, the downregulation of these secondary metabolites seems to affect the growth of *S. mutans*.

## Discussion

Diterpenoids derived from *A. continentalis* roots exhibit antibacterial activity against several bacteria (Wilkens et al., 2002; Jeong et al., 2006, 2008, 2010, 2013; Martins et al., 2018). CA and KA are particularly known to inhibit the growth, acid production, biofilm formation, and the adherence of *S. mutans* at a low concentration of 4 μg/ml (Jeong et al., 2010, 2013). AA, which was first isolated from *A. continentalis*, is also known to exert antibacterial activity against *S. mutans* (Kim, 2006). Urzúa et al. suggested that a hydrophobic moiety with a substituted decalin skeleton and a hydrophilic region with one hydrogen-bond-donor group of a diterpenoid are essential for the antibacterial activity (Urzúa et al., 2008). AA, CA, and KA meet two structural requirements proposed by Urzúa et al. Wilkens reported that the antibacterial activity of KA is associated with the cell membrane. Therefore, this suggests that the antibacterial activity differs depending on whether the bacteria are Gram-positive or Gram-negative. KA showed bacteriolytic activity against *B. cereus* but showed resistance to LPS mutants of *Salmonella typhi.* However, the spheroplasts of *Escherichia coli* were more sensitive to KA (Wilkens et al., 2002).

In this study, the morphology of *S. mutans* was altered when cells were exposed to 4 μg/ml of DM. The extracellular coating of the bacteria disappeared at a concentration of 40 μg/ml of DM. However, this strain was found to regulate the fatty acid composition of cell membranes to protect cells from environmental changes caused by external compounds. Willdigg et al. reported that the membrane stress response allows bacteria to modify cell membranes by regulating the length, branching and saturation of fatty acid acyl chains, altering membrane lipid composition, or synthesizing proteins that modify or protect the membrane (Willdigg and Helmann, 2021). *Streptococcus mutans* can survive acidic environments by altering the membrane fatty acids from the short-chained and saturated membrane fatty acids to the long-chained and unsaturated fatty acids (Fozo and Quivey, 2004). *Escherichia coli* and *S. aureus* were also found to produce more unsaturated fatty acids in the presence of naringenin (Wang et al., 2018). *Chryseobacterium frigidisoli* caused an increase in the anteiso-and bis-unsaturated fatty acid content at low temperatures. However, it increased long-chain unsaturated *iso*- and *anteiso*-fatty acid content at low pH (Bajerski et al., 2017). According to these reports, the unsaturated fatty acid composition in DM-treated *S. mutans* increased to a larger extent than that in untreated cells. Transcriptome analysis confirmed the rationale for changes in cells induced by diterpenoid treatment. The genes involved in fatty acid synthesis were downregulated by CA, whereas *fabM* was relatively upregulated by AA compared with untreated cells. *fabM* is the gene that is encoded for the biosynthesis of monounsaturated fatty acids. Changes in the fatty acid composition based on the membrane fatty acid composition analysis and the DEGs of fatty acid synthesis genes following DM treatment possibly affect the bacterial survival and morphology (Fozo et al., 2007).

Diterpenoids also affected the gene expression associated with cell division and envelope biogenesis. The recruitment and polymerization of FtsZ initiate cell division to form a Z-ring at mid-cell marked by a microscopically visible “equatorial ring.” Furthermore, several membrane-associated division proteins are recruited to form the divisome and trigger peptidoglycan (PG) biosynthesis and chromosome segregation (Wang et al., 2020). In this process, *ftsX* hydrolyzes septal PG when the cell wall is split. While *ftsW* contributes to PG synthesis as one of the PG synthase complexes (PBP1b-FtsW-FtsI), *ftsL* acts as a PG synthesis inhibitor (Wang et al., 2020). The irregular morphology of *S. mutans* by DM appears to be caused by the downexpression of *ftsWXZ* and upexpression of *ftsL.* Xiang et al. reported that an *ezrA* mutant of *S. mutans* was slow-growing with a shorter length, extended width, and rounded cell shape than the wild-type (Xiang et al., 2019). Land et al. showed that the depletion of MreCD in *Streptococcus pneumoniae* resulted in cell rounding and lysis (Land and Winkler, 2011). Downregulation of *ezrA* and *mreCD* by diterpenoids likely affects the irregular cell shape of *S. mutans*. Furthermore, Brown et al. also reported that PG layers of Gram-positive bacteria were densely functionalized with anionic glycopolymers. The anionic glycopolymers are also called wall teichoic acids (WTAs), which significantly contribute to cell shape determination, cell division regulation, other fundamental aspects of bacterial physiology, pathogenesis, and antibiotic resistance (Brown et al., 2013). The product of the *dltXABCD* operon mediates alanylation of WTA, which reduces the negative charge of the cell envelope to prevent cationic antimicrobial peptides (Kamar et al., 2017; Wood et al., 2018). RGPs, which are functionally homologous to WTAs, distinctly contribute to protecting *S. mutans* from various stress conditions associated with pathogenicity and maintaining morphology (Kovacs et al., 2019). Our transcriptome data showed that *S. mutans* possibly upregulates both genes that synthesize WTAs and RGPs to counteract the antimicrobial activity of diterpenoids. A Cid/Lrg system is involved in autolysis, biofilm formation, glucosyltransferase expression, and oxidative stress resistance in *S. mutans* (Ahn et al., 2010). The downregulation of *cidAB* and *lrgAB* by diterpenoids can affect cell viability by decreasing the expression of glucosyltransferase and biofilm formation-related genes, which is consistent with the inhibition of cell growth.

Bacteria adapt to environmental fluctuations by rapidly sensing and regulating gene expression through TCSs. So far, 15 sets of TCSs have been predicted in *S. mutans* UA159 (Ajdić et al., 2002; Kawada-Matsuo and Komatsuzawa, 2017). Among the predicted TCSs, *lia* operon responds to cell membrane-disrupting antibacterial agents and regulates cell envelope biogenesis, chaperone, protease, and transcription factors (Suntharalingam et al., 2009). *bceABRS* and *lcrSR* are associated with bacitracin and nukacin resistance, respectively (Ouyang et al., 2010). Diterpenoids were found to upregulate the three TCSs mentioned above. Additionally, *murB*, *htrA*, SMU.753, and *spxA* regulated by LiaSR and SMU.862 regulated by BceRS were also upregulated.

Lee et al. suggested that genetic mutations in *S. mutans* fluoride-resistant strains resulted in the reduced activity of PTSs, such as ManXYZ and ManLMN, which strongly affect carbon inflow and energy production *via* the central carbon metabolism, whereas the upregulation of MsmEFGK and MalXFGK can overcome the restricted carbon flow (Lee et al., 2021). Transcriptomic analyses demonstrated that diterpenoids downregulated genes associated with most PTSs. Sugar alcohol and trehalose PTSs and oligosaccharide permeases were unaffected. Furthermore, diterpenoids also reduced the expression of genes involved in glycolysis and carbohydrate metabolism. These results indicate that the absorption and metabolism of carbohydrates and energy production were decreased as a stress response to diterpenoids. These reductions affect the downregulation of nucleotide, fatty acid, and amino acid metabolism. By upregulating sugar alcohol and trehalose PTS, *S. mutans* can overcome limited carbohydrate intake. This finding also corroborates the results demonstrated by Lee et al. Also, given that they are more reduced chemically, these compounds have higher energy than their analogous sugar. Mannitol also functions as an antioxidant reagent (Williamson et al., 2002).

Coenzyme A functions as an acyl carrier and carbonyl-activating group in numerous reactions central to cellular metabolism. It also provides a 4′-phosphopantethane prosthetic group integrated by a carrier protein, which plays an important role in the biosynthesis of fatty acids, polyketides, and non-ribosomal peptides (Spry et al., 2008). Pantothenic acid, a precursor of coenzyme A, is essential for the growth of many lactic acid bacteria (Snell et al., 1938). Most amino acid metabolism and permease-related genes in *S. mutans* were downregulated by diterpenoid treatment, whereas cysteine-related genes were highly upregulated. In the pathway for pantothenic acid to coenzyme A, cysteine reacts with 4′-phosphopantothenate to form 4′-phosphophantane (Spry et al., 2008). The upregulation of coenzyme A synthesis can also overcome the downregulation of various metabolisms by diterpenoids. This is similar to the consequences of carbohydrate metabolism.

## Conclusion

Three types of diterpenoids, AA, CA, and KA were extracted from *A. continentalis.* Their antibacterial activities against *S. mutans* were examined. When *S. mutans* was exposed to diterpenoids, the bacterium demonstrated an irregular shape. In addition, the proportion of the unsaturated fatty acids was relatively increased compared with saturated fatty acids. Transcriptome analysis showed that DEGs of *S. mutans* after diterpenoid treatment exhibited a similar pattern, notwithstanding the diterpenoid used in this study. This suggests that the antibacterial mechanism induced by each diterpenoid is universal. The diterpenoid treatment mainly affected cell membrane synthesis, cell division, and carbohydrate uptake and metabolism in *S. mutans*. The restricted carbohydrate intake and inhibition of glycolytic pathways appear to decrease the energy production in *S. mutans*. It also affects the metabolism of amino acid, nucleotide, and fatty acid biosynthesis. *Streptococcus mutans* caused the upregulation of sugar alcohol transporters, cysteine-related genes, coenzyme A synthesis genes, and cell envelope stress response-related TCSs in response to diterpenoids. This is the first study to provide information on the antibacterial effects of diterpenoids on *S. mutans* and its underlying mechanism.

## Data availability statement

The datasets presented in this study can be found in online repositories. The names of the repository/repositories and accession number(s) can be found at: https://www.ncbi.nlm.nih.gov/, GSE202812.

## Author contributions

KM and H-JL performed experiments. SH and EJ provided sequence data and bioinformatic analysis. KM and SH contributed to data analysis and wrote first draft of the manuscript. JNK and JC supervised the work. KM, JNK, and JC contributed to the preparation of the final article. All authors contributed to the study conception and design. All authors contributed to the article and approved the submitted version.

## Funding

This work was supported by Basic Science Research Program through the National Research Foundation of Korea (NRF) funded by the Ministry of Science, ICT & Future Planning (2020R1A2C1003388) to JNK, and the Ministry of Education (2020R1I1A1A01073109) to KM.

## Conflict of interest

The authors declare that the research was conducted in the absence of any commercial or financial relationships that could be construed as a potential conflict of interest.

## Publisher’s note

All claims expressed in this article are solely those of the authors and do not necessarily represent those of their affiliated organizations, or those of the publisher, the editors and the reviewers. Any product that may be evaluated in this article, or claim that may be made by its manufacturer, is not guaranteed or endorsed by the publisher.

## Footnotes

^1^ https://www.bioinformatics.babraham.ac.uk/projects/fastqc

^2^ https://www.ncbi.nlm.nih.gov/genome/856?genome_assembly_id=169364
