# Supplementary material for: Identification of the antibacterial action mechanism of diterpenoids through transcriptome profiling
Source: Front Microbiol. 2022 Jul 26;13:945023. doi: 10.3389/fmicb.2022.945023 (PMC9360744; doi:10.3389/fmicb.2022.945023)
Supplement: Supplementary file 1 [file Data_Sheet_1.docx]

| Carbon No | Acanthoic acid | | Continentalic acid | | Kaurenoic acid | |
| --- | --- | --- | --- | --- | --- | --- |
|  | δ_C_ | δ_H_ | δ_C_ | δ_H_, | δ_C_ | δ_H_ |
| 1 | 42.15 | 1.27–1.24, 1H, m  1.02–0.99, 1H, m | 39.42 |  | 40.91 |  |
| 2 | 20.58 | 1.93–1.85, 1H, m  1.48–1.43, 1H, m | 19.42 |  | 19.30 |  |
| 3 | 38.40 | 2.17–2.12, 1H, m  1.04–1.00, 1H, m | 38.17 |  | 38.02 |  |
| 4 | 44.41 |  | 44.23 |  | 44.06 |  |
| 5 | 48.21 | 1.64, 1H, dd, *J* = 4.0, 13.2 Hz | 56.27 |  | 57.25 |  |
| 6 | 19.19 | 2.22–2.15, 1H, m  1.96–1.89, 1H, m | 24.31 |  | 22.04 |  |
| 7 | 27.99 | 1.73–1.70, 1H, m  1.25–1.21, 1H, m | 35.99 |  | 41.48 |  |
| 8 | 28.92 | 2.34–2.27, 1H, m | 138.17 |  | 44.44 |  |
| 9 | 150.10 |  | 50.71 |  | 55.31 |  |
| 10 | 38.67 |  | 39.40 |  | 39.91 |  |
| 11 | 116.86 | 5.38, 1H, dd, *J* = 3.6, 5.4 Hz | 19.77 |  | 18.64 |  |
| 12 | 37.73 | 2.02, 1H, br. d, *J* = 19.2 Hz  1.75–1.72, 1H, m | 36.61 |  | 33.32 |  |
| 13 | 35.09 |  | 38.72 |  | 43.93 | 2.62, 1H, br.s |
| 14 | 42.05 | 1.77, 1H, br. d, *J* = 13.2 Hz  1.42, 1H, dd, *J* = 4.0, 13.2 Hz | 128.18 | 5.13,1H, br. s | 39.87 |  |
| 15 | 150.48 | 5.80, 1H, dd, *J* = 10.8, 17.4 Hz | 147.39 | 5.69, 1H, dd, *J* = 10.5, 17.1 Hz | 49.17 |  |
| 16 | 109.39 | 4.91, 1H, dd, *J* = 1.8, 17.4 Hz  4.85, 1H, dd, *J* = 1.8, 10.8 Hz | 113.10 | 4.92,1H, dd, *J* = 1.8,10.5Hz  4.89,1H, dd, *J* = 1.8, 17.1 Hz | 156.14 |  |
| 17 | 22.42 | 0.93, 3H, s | 29.55 | 0.98, 3H, s | 103.20 | 4.78, 1H, br.s  4.72, 1H, br.s |
| 18 | 28.79 | 1.23, 3H, s | 29.37 | 1.24, 3H, s | 29.18 | 1.22, 3H, s |
| 19 | 184.43 |  | 184.10 |  | 184.23 |  |
| 20 | 22.65 | 0.98, 3H, s | 14.01 | 0.62, 3H, s | 15.81 | 0.93, 3H, s |

Supplementary Table 1. NMR data of three major diterpenoids in *A. continentalis*

Supplementary Table 2. Differential gene expression associated with biofilm formation

| Locus ID | Annotation | Gene | Gene expression (log_2_ FC)* | | | |
| --- | --- | --- | --- | --- | --- | --- |
|  |  |  | AA | CA | KA | DM |
| SMU_RS04620 | glucosyltransferase-I | *gtfB* | -0.77 | -2.41 | -1.93 | -1.81 |
| SMU_RS04625 | glucosyltransferase-SI | *gtfC* | -1.11 | -3.10 | -2.81 | -2.57 |
| SMU_RS04210 | glucosyltransferase-S | *gtfD* | -0.51 |  | 0.78 | 0.70 |
| SMU_RS01995 | LCP family protein | *brpA* | 0.32 | 0.50 | 0.49 | 0.51 |
| SMU_RS00190 | CHAP domain-containing protein | *gbpB* | -1.46 | -2.03 | -2.11 | -2.23 |
| SMU_RS09345 | bifunctional (p)ppGpp synthetase/guanosine-3',5'-bis(diphosphate) 3'-pyrophosphohydrolase |  |  |  |  |  |
| SMU_RS02900 | cell surface antigen I/II | *spaP* |  |  |  |  |
| SMU_RS06885 | response regulator transcription factor | *covR* | -0.81 |  |  |  |

* Gene expression is expressed using absolute log_2_ fold change (FC) values with adjusted *p*-values less than 0.05. Red and blue correspond to up- or down-regulated gene expression levels, respectively.

Supplementary Table 3. Differential gene expression associated with coenzyme metabolism

| Locus ID | Annotation | Gene | Gene expression (log_2_ FC)* | | | |
| --- | --- | --- | --- | --- | --- | --- |
|  |  |  | AA | CA | KA | DM |
| SMU_RS00475 | bifunctional hydroxymethylpyrimidine kinase/phosphomethylpyrimidine kinase | *thiD* | -1.21 | -1.36 | -1.44 | -1.45 |
| SMU_RS00590 | 1-phosphofructokinase | *fruP* | 1.01 | 2.16 | 2.91 | 2.69 |
| SMU_RS01195 | ketol-acid reductoisomerase | *ilvC* | -0.58 | -1.29 | -1.40 | -1.47 |
| SMU_RS01350 | bifunctional glutamate--cysteine ligase GshA/glutathione synthetase | *gshAB* | 0.39 | 0.42 | 0.30 | 0.38 |
| SMU_RS01470 | 2-dehydropantoate 2-reductase |  | -1.43 | -2.18 | -2.55 | -2.65 |
| SMU_RS01560 | hypothetical protein |  | 1.54 | 2.39 | 2.47 | 2.56 |
| SMU_RS01855 | aminotransferase class III-fold pyridoxal phosphate-dependent enzyme |  |  | 2.23 | 1.99 | 2.11 |
| SMU_RS02235 | nicotinate phosphoribosyltransferase |  |  | -0.62 | -0.52 | -0.57 |
| SMU_RS02240 | ammonia-dependent NAD(+) synthetase | *nadE* |  |  | 0.44 | 0.48 |
| SMU_RS02285 | S-ribosylhomocysteine lyase | *luxS* |  | -1.56 | -1.96 | -1.84 |
| SMU_RS02370 | fructose-6-phosphate aldolase |  | 0.68 | 0.87 | 1.50 | 0.81 |
| SMU_RS02490 | pantetheine-phosphate adenylyltransferase | *coaD* | 0.99 | 1.68 | 1.86 | 1.97 |
| SMU_RS03355 | phosphoenolpyruvate carboxylase | *ppc* |  | 1.41 | 1.34 | 1.51 |
| SMU_RS03890 | bifunctional folylpolyglutamate synthase/dihydrofolate synthase | *fol* |  | 1.47 | 0.63 | 0.71 |
| SMU_RS03905 | tRNA 4-thiouridine(8) synthase | *thiI* | 0.67 | 1.20 | 1.27 | 1.28 |
| SMU_RS04235 | 7-carboxy-7-deazaguanine synthase | *queE* | -0.68 |  |  |  |
| SMU_RS04240 | 6-carboxytetrahydropterin synthase |  |  | 1.94 | 1.66 | 1.83 |
| SMU_RS04300 | uroporphyrinogen decarboxylase |  | 3.66 | 4.48 | 5.90 | 4.50 |
| SMU_RS04365 | permease |  |  |  | -0.32 |  |
| SMU_RS04370 | dihydrofolate reductase | *dfrA* | 0.72 | 1.10 | 1.23 | 1.32 |
| SMU_RS04400 | pyridoxamine kinase |  | -1.58 | -2.13 | -1.87 | -2.07 |
| SMU_RS04465 | dihydroneopterin aldolase | *folA* | 0.81 | 1.48 | 1.72 | 1.80 |
| SMU_RS04470 | 2-amino-4-hydroxy-6-hydroxymethyldihydropteridine diphosphokinase | *folK* | 0.77 | 1.41 | 1.61 | 1.66 |
| SMU_RS04655 | triphosphoribosyl-dephospho-CoA synthase | *citG* | 0.86 | 1.62 | 1.73 | 1.87 |
| SMU_RS04700 | citrate lyase holo-[acyl-carrier protein] synthase | *citG2* | 1.88 | 2.29 | 3.19 | 3.52 |
| SMU_RS04815 | NAD kinase | *nadK* | 0.88 | 1.57 | 1.39 | 1.62 |
| SMU_RS04950 | phosphopantothenate--cysteine ligase |  | 1.65 | 3.40 | 3.12 | 3.21 |
| SMU_RS04955 | phosphopantothenoylcysteine decarboxylase | *coaG* | 1.91 | 2.06 | 2.03 | 2.19 |
| SMU_RS05020 | FAD:protein FMN transferase | *apbE* | 1.21 | 1.19 | 1.58 | 1.82 |
| SMU_RS05265 | bifunctional riboflavin kinase/FAD synthetase |  | 0.89 | 1.26 | 1.55 | 1.66 |
| SMU_RS06130 | mutanobactin A biosynthesis phosphopantetheinyl transferase | *sfp* |  | 1.52 |  |  |
| SMU_RS06325 | phosphoenolpyruvate carboxykinase (ATP) | *pckA* |  | -1.89 | -1.26 | -1.29 |
| SMU_RS06600 | acetolactate synthase | *alsS* |  | -0.99 | -1.24 | -1.22 |
| SMU_RS06785 | tagatose-6-phosphate kinase | *lacC* | 0.94 |  |  |  |
| SMU_RS06985 | glucose-1-phosphate adenylyltransferase | *glgC* | 1.35 | -0.75 | 0.79 | 0.90 |
| SMU_RS07135 | methionine adenosyltransferase | *metK* | -1.58 | -3.11 | -3.01 | -3.23 |
| SMU_RS07160 | bifunctional biotin--[acetyl-CoA-carboxylase] synthetase/biotin operon repressor | *birA* |  | -0.49 | -0.74 | -0.76 |
| SMU_RS07315 | class I SAM-dependent methyltransferase |  | 0.66 | 0.41 | 0.73 | 0.75 |
| SMU_RS07320 | dephospho-CoA kinase | *coaE* | 1.78 | 1.79 | 2.11 | 2.07 |
| SMU_RS07505 | D-3-phosphoglycerate dehydrogenase | *serA* | 0.89 | 1.22 | 1.30 | 1.25 |
| SMU_RS07530 | 16S rRNA (cytidine(1402)-2'-O)-methyltransferase | *rsmI* | 0.76 | 0.79 | 0.79 | 0.94 |
| SMU_RS08200 | isoprenyl transferase | *uppS* | -1.13 | -1.60 | -1.80 | -1.72 |
| SMU_RS08250 | HD domain-containing protein |  | 2.05 | 3.51 | 3.71 | 3.86 |
| SMU_RS08255 | nicotinate-nucleotide adenylyltransferase | *nadD* | 1.67 | 2.35 | 2.51 | 2.65 |
| SMU_RS09115 | gamma-glutamylcysteine synthetase |  |  | 1.15 | 0.60 | 0.66 |
| SMU_RS09435 | ferrochelatase | *cpfC* | -0.72 | -0.96 | -0.87 | -0.72 |

* Gene expression is expressed using absolute log_2_ fold change (FC) values with adjusted *p*-values less than 0.05. Red and blue correspond to up- or down-regulated gene expression levels, respectively.

Supplementary Table 4. Differential gene expression associated with amino acid transport and metabolism

| Locus ID | Annotation | Gene | Gene expression (log_2_ FC)* | | | |  |
| --- | --- | --- | --- | --- | --- | --- | --- |
|  |  |  | AA | CA | KA | DM | |
| SMU_RS00070 | amino acid permease |  | -2.12 | -2.45 | -2.58 | -2.97 | |
| SMU_RS00200 | pyridoxal phosphate-dependent aminotransferase |  | 1.11 | 0.98 | 0.84 | 1.05 | |
| SMU_RS00315 | ATP-grasp domain-containing protein |  | -0.50 |  | -0.83 | -1.10 | |
| SMU_RS00405 | threonine synthase | *thrC* | -0.48 | -1.13 | -1.25 | -1.37 | |
| SMU_RS00675 | amidohydrolase |  |  | -0.78 |  | -0.85 | |
| SMU_RS00680 | MFS transporter |  |  | -2.56 | -2.22 | -2.30 | |
| SMU_RS01190 | acetolactate synthase small subunit | *ilvH* | -0.97 | -1.00 | -1.22 | -1.35 | |
| SMU_RS01230 | amino acid ABC transporter ATP-binding protein |  | -1.63 | -1.90 | -1.69 | -1.76 | |
| SMU_RS01270 | cysteine desulfurase | *nifS* | 0.49 | 0.39 | 0.55 | 0.56 | |
| SMU_RS01305 | ABC transporter permease |  | -1.53 | -2.23 | -1.99 | -2.10 | |
| SMU_RS01330 | putrescine carbamoyltransferase | *ptcA* | 1.23 | 1.27 | 1.51 | 1.52 | |
| SMU_RS01565 | 2,3,4,5-tetrahydropyridine-2,6-dicarboxylate N-acetyltransferase | *dapH* | -1.16 | -1.86 | -2.09 | -2.10 | |
| SMU_RS01570 | N-acetyldiaminopimelate deacetylase |  | -0.90 | -1.50 | -1.65 | -1.63 | |
| SMU_RS01640 | argininosuccinate synthase | *argG* | -1.02 | -1.01 | -0.79 | -0.88 | |
| SMU_RS01645 | argininosuccinate lyase | *argH* | -1.59 | -2.42 | -2.25 | -2.27 | |
| SMU_RS01795 | type I glutamate--ammonia ligase | *glnA* |  | -2.01 | -2.04 | -1.55 | |
| SMU_RS01800 | glutamate synthase large subunit | *gltA* | -0.53 | -1.72 | -1.63 | -1.54 | |
| SMU_RS01805 | glutamate synthase subunit beta | *gltB* | -0.63 | -1.71 | -1.47 | -1.33 | |
| SMU_RS01900 | AzlC family ABC transporter permease |  | -1.48 | -2.12 | -2.11 | -2.23 | |
| SMU_RS01930 | Xaa-Pro dipeptidyl-peptidase | *pepX* | -0.37 |  |  |  | |
| SMU_RS01940 | peptidase |  | 1.57 | 3.31 | 2.93 | 3.04 | |
| SMU_RS02210 | transporter substrate-binding domain-containing protein |  | 1.15 | 4.13 | 4.53 | 3.73 | |
| SMU_RS02220 | amino acid ABC transporter ATP-binding protein |  | -0.81 | -0.88 |  | -1.23 | |
| SMU_RS02550 | chorismate mutase |  | -1.75 | -2.12 | -2.10 | -2.26 | |
| SMU_RS02570 | indole-3-glycerol phosphate synthase | *trpC* | -0.60 | -0.57 | -0.51 | -0.45 | |
| SMU_RS02575 | phosphoribosylanthranilate isomerase | *trpF* |  | 1.24 | 1.37 | 1.56 | |
| SMU_RS02585 | tryptophan synthase subunit alpha | *trpA* |  | 0.46 | 0.72 | 0.86 | |
| SMU_RS02695 | ornithine carbamoyltransferase | *argF* | -0.89 |  |  | -0.45 | |
| SMU_RS02715 | amino acid ABC transporter ATP-binding protein |  | -0.92 | -1.25 | -1.11 | -1.31 | |
| SMU_RS02800 | SGNH/GDSL hydrolase family protein |  | 0.83 | 1.75 | 1.40 | 1.62 | |
| SMU_RS03055 | oligoendopeptidase F | *pepB* | 1.71 | 2.34 | 2.23 | 2.37 | |
| SMU_RS03145 | bifunctional ornithine acetyltransferase/N-acetylglutamate synthase | *argJ* |  | -0.38 |  |  | |
| SMU_RS03155 | acetylornithine transaminase | *argD* | -0.90 | -1.96 | -1.82 | -1.82 | |
| SMU_RS03495 | DHA2 family efflux MFS transporter permease subunit | *lmrB* | -2.09 | -2.75 | -2.90 | -3.04 | |
| SMU_RS03615 | type I 3-dehydroquinate dehydratase | *aroD* | 0.85 | 1.86 | 2.19 | 2.33 | |
| SMU_RS03620 | shikimate dehydrogenase | *aroE* | 0.76 | 1.48 | 1.89 | 2.00 | |
| SMU_RS03625 | 3-dehydroquinate synthase | *aroB* |  |  | 0.48 | 0.61 | |
| SMU_RS03630 | chorismate synthase | *aroC* |  | -0.42 |  |  | |
| SMU_RS03635 | prephenate dehydrogenase |  |  | 0.45 | 0.88 | 1.03 | |
| SMU_RS03645 | 3-phosphoshikimate 1-carboxyvinyltransferase | *aroA* | 0.42 |  |  |  | |
| SMU_RS03735 | amino acid ABC transporter ATP-binding protein |  |  | -2.66 | -2.54 | -2.22 | |
| SMU_RS03775 | aminotransferase |  | -0.54 | -1.14 | -1.54 | -1.46 | |
| SMU_RS03780 | transporter substrate-binding domain-containing protein |  |  | 1.41 | 0.97 | 1.12 | |
| SMU_RS04180 | 4-hydroxy-tetrahydrodipicolinate reductase | *dapB* | -0.89 | -2.28 | -2.04 | -2.05 | |
| SMU_RS04220 | NADP-specific glutamate dehydrogenase |  | -0.73 | -1.51 | -2.01 | -2.00 | |
| SMU_RS04305 | amino acid ABC transporter substrate-binding protein |  | 3.59 | 4.82 | 6.41 | 4.99 | |
| SMU_RS04320 | amino acid ABC transporter ATP-binding protein |  | 2.41 | 2.42 | 4.08 | 2.67 | |
| SMU_RS04385 | amino acid permease |  | -0.56 | -2.15 | -1.88 | -1.92 | |
| SMU_RS04445 | homoserine kinase | *thrB* |  | -0.39 | -0.32 | -0.32 | |
| SMU_RS04550 | aspartate-semialdehyde dehydrogenase | *asd* | -0.58 | -1.30 | -1.21 | -1.31 | |
| SMU_RS04555 | 4-hydroxy-tetrahydrodipicolinate synthase | *dapA* | -0.83 | -1.72 | -1.57 | -1.69 | |
| SMU_RS04840 | cysteine desulfurase |  | 1.71 | 2.57 | 2.56 | 2.66 | |
| SMU_RS04860 | JAB domain-containing protein | *radC* | -0.81 | -1.42 | -1.02 | -1.44 | |
| SMU_RS04890 | ABC transporter permease/substrate binding protein | *opuAb* |  | -1.72 | -1.54 | -1.22 | |
| SMU_RS04895 | glycine betaine/L-proline ABC transporter ATP-binding protein | *opuAa* | 0.45 | -0.44 |  |  | |
| SMU_RS04990 | serine hydroxymethyltransferase | *glyA* |  | -1.28 | -0.88 | -0.76 | |
| SMU_RS05055 | ABC transporter ATP-binding protein | *opuBa* |  |  | -0.96 |  | |
| SMU_RS05095 | hypothetical protein |  | 0.99 | 2.99 | 2.33 | 2.49 | |
| SMU_RS05115 | VOC family protein |  |  | 1.88 | 1.68 | 1.93 | |
| SMU_RS05210 | M1 family metallopeptidase | *pepN* | 0.51 | 0.62 | 0.59 | 0.77 | |
| SMU_RS05400 | O-acetylhomoserine aminocarboxypropyltransferase/cysteine synthase | *cysD* | 0.79 | 0.41 | 0.86 | 1.04 | |
| SMU_RS05410 | sodium:alanine symporter family protein |  |  |  | -1.26 | -1.20 | |
| SMU_RS05420 | amino acid ABC transporter substrate-binding protein |  |  | 1.30 | 1.11 | 1.31 | |
| SMU_RS05610 | transporter substrate-binding domain-containing protein |  |  | 1.33 | 1.59 | 1.64 | |
| SMU_RS05720 | dipeptidase PepV | *pepV* | 0.88 |  |  |  | |
| SMU_RS05820 | phosphoribosyl-AMP cyclohydrolase | *hisI* |  |  |  | 0.65 | |
| SMU_RS05825 | imidazole glycerol phosphate synthase subunit HisF | *hisF* |  | -1.13 | -0.96 | -0.87 | |
| SMU_RS05830 | 1-(5-phosphoribosyl)-5-[(5-phosphoribosylamino)methylideneamino]imidazole-4-carboxamide isomerase | *hisA* |  | -0.47 |  |  | |
| SMU_RS05835 | imidazole glycerol phosphate synthase subunit | *hisH* |  | -1.00 | -0.96 | -0.81 | |
| SMU_RS05845 | imidazoleglycerol-phosphate dehydratase | *hisB* |  | -1.15 | -1.10 | -0.94 | |
| SMU_RS05850 | phosphoserine phosphatase | *serB* |  | -0.84 | -0.85 | -0.68 | |
| SMU_RS05855 | histidinol dehydrogenase | *hisD* |  | -0.95 | -0.93 | -0.82 | |
| SMU_RS05865 | ATP phosphoribosyltransferase regulatory subunit | *hisZ* | 0.46 | 0.41 |  | 0.43 | |
| SMU_RS05870 | histidinol-phosphate transaminase | *hisC* |  |  | -0.44 |  | |
| SMU_RS05915 | MFS transporter |  | -2.49 | -2.84 | -3.52 | -3.61 | |
| SMU_RS06000 | C69 family dipeptidase |  | 0.85 | 0.48 | 0.64 | 0.79 | |
| SMU_RS06040 | pyridoxal phosphate-dependent aminotransferase | *aspB* |  | 0.90 | 1.15 | 1.27 | |
| SMU_RS06150 | mutanobactin A system MFS transporter |  |  | -1.05 | -1.65 | -1.82 | |
| SMU_RS06290 | 3-isopropylmalate dehydratase small subunit | *leuD* | 1.14 | 1.82 | 2.07 | 2.02 | |
| SMU_RS06305 | 2-isopropylmalate synthase | *leuA* |  | -0.56 | -0.77 | -0.78 | |
| SMU_RS06590 | amino acid permease |  | -1.86 | -2.45 | -2.53 | -2.59 | |
| SMU_RS06600 | acetolactate synthase | *alsS* |  | -0.99 | -1.24 | -1.22 | |
| SMU_RS06635 | FAD-binding oxidoreductase |  | 0.57 | 0.53 | 1.06 | 1.27 | |
| SMU_RS06730 | GNAT family N-acetyltransferase |  | -0.33 | -0.93 | -0.83 | -0.76 | |
| SMU_RS06745 | hypothetical protein |  | -0.80 | -1.42 | -1.65 | -1.53 | |
| SMU_RS06750 | cobalamin-independent methionine synthase II family protein |  | -1.45 | -1.98 | -1.99 | -1.98 | |
| SMU_RS06890 | amino acid ABC transporter ATP-binding protein | *glnQ* |  | -1.27 | -1.89 | -1.24 | |
| SMU_RS07140 | haloacid dehalogenase-like hydrolase |  |  | 1.83 | 1.10 | 1.25 | |
| SMU_RS07220 | aminopeptidase P family protein | *pepQ* | -1.08 | -0.56 | -0.92 | -0.81 | |
| SMU_RS07275 | lactoylglutathione lyase | *lguL* | 1.18 | 0.66 |  |  | |
| SMU_RS07285 | DHA2 family efflux MFS transporter permease subunit |  |  | -0.61 | -0.63 | -0.62 | |
| SMU_RS07310 | multidrug efflux MFS transporter |  | -2.00 | -3.13 | -2.68 | -2.85 | |
| SMU_RS07410 | 5'-methylthioadenosine/adenosylhomocysteine nucleosidase | *pfs* | -0.64 | -0.80 | -0.86 | -0.76 | |
| SMU_RS07505 | D-3-phosphoglycerate dehydrogenase | *serA* | 0.89 | 1.22 | 1.30 | 1.25 | |
| SMU_RS07515 | 3-phosphoserine/phosphohydroxythreonine transaminase | *serC* | -0.58 | -1.50 | -1.63 | -1.73 | |
| SMU_RS07560 | ABC transporter ATP-binding protein | *livF* | -0.50 | -0.86 | -0.63 | -0.69 | |
| SMU_RS07570 | branched-chain amino acid ABC transporter permease | *livM* | -1.06 | -2.00 | -1.89 | -1.98 | |
| SMU_RS07575 | branched-chain amino acid ABC transporter permease | *livH* | -1.20 | -2.16 | -2.08 | -2.21 | |
| SMU_RS07605 | pyridoxal phosphate-dependent aminotransferase | *metC* | 1.55 | 3.14 | 3.60 | 3.40 | |
| SMU_RS07610 | cystathionine gamma-synthase | *metB* | 1.23 | 1.96 | 2.20 | 1.97 | |
| SMU_RS07640 | VOC family protein |  |  | -0.88 | -1.17 | -0.98 | |
| SMU_RS07820 | diaminopimelate decarboxylase |  | -0.71 | -1.59 | -1.50 | -1.55 | |
| SMU_RS08290 | EamA family transporter |  | -1.55 | -2.00 | -2.31 | -2.40 | |
| SMU_RS08410 | 3-deoxy-7-phosphoheptulonate synthase | *aroG* |  | 1.26 | 1.33 | 1.42 | |
| SMU_RS08415 | 3-deoxy-7-phosphoheptulonate synthase | *aroH* |  | 1.26 | 1.29 | 1.40 | |
| SMU_RS08475 | aminopeptidase P family protein | *pepP* | 0.72 | 1.01 | 1.21 | 1.30 | |
| SMU_RS08545 | zinc-dependent alcohol dehydrogenase family protein |  |  | -0.59 | -0.98 | -1.07 | |
| SMU_RS08820 | M20/M25/M40 family metallo-hydrolase |  | -0.85 | -0.47 |  | -0.42 | |
| SMU_RS08830 | amino acid ABC transporter substrate-binding protein |  |  | 1.44 | 1.51 | 1.05 | |
| SMU_RS08975 | pyrroline-5-carboxylate reductase | *proC* | -0.67 | -1.38 | -1.41 | -1.45 | |
| SMU_RS09615 | APC family permease |  | -2.16 | -2.71 | -2.93 | -3.39 | |
| SMU_RS09645 | multidrug efflux MFS transporter |  | -2.39 | -3.20 | -3.00 | -3.39 | |
| SMU_RS09675 | betaine/proline/choline family ABC transporter ATP-binding protein | *opuCa* | 0.98 | 1.01 | 0.95 | 1.19 | |
| SMU_RS09725 | dihydroxy-acid dehydratase | *ilvD* | -0.75 | -2.53 | -2.21 | -2.26 | |

Supplementary Table 5. Differential gene expression associated with secondary metabolite biosynthesis, transport, and catabolism

| Locus ID | Annotation | Gene | Gene expression (log_2_ FC)* | | | |
| --- | --- | --- | --- | --- | --- | --- |
|  |  |  | AA | CA | KA | DM |
| SMU_RS00215 | acyl carrier protein | *acpP* | 0.90 | 2.91 | 2.83 | 2.93 |
| SMU_RS01530 | SDR family oxidoreductase |  |  | -1.84 | -1.37 | -1.45 |
| SMU_RS01590 | NAD(P)H-dependent glycerol-3-phosphate dehydrogenase | *gpsA* |  | -0.74 | -0.84 | -0.75 |
| SMU_RS01600 | cysteine hydrolase |  | -0.54 | -0.93 | -0.88 | -0.94 |
| SMU_RS01845 | SDR family oxidoreductase |  | 1.31 | 3.16 | 2.74 | 3.10 |
| SMU_RS03680 | PaaI family thioesterase |  | 1.14 | 1.54 | 1.52 | 1.63 |
| SMU_RS05990 | class I SAM-dependent methyltransferase |  |  | 0.92 | 0.59 | 0.66 |
| SMU_RS06085 | (S)-acetoin forming diacetyl reductase | *budC* | 1.06 |  |  |  |
| SMU_RS06155 | non-ribosomal peptide synthetase | *bacD* | -3.45 | -3.06 | -3.21 | -3.77 |
| SMU_RS06160 | non-ribosomal peptide synthetase | *bacA2* | -3.79 | -2.93 | -3.27 | -3.65 |
| SMU_RS06165 | mutanobactin A non-ribosomal peptide synthetase MubB |  | -3.66 | -3.17 | -3.23 | -3.63 |
| SMU_RS06170 | non-ribosomal peptide synthetase | *bacA1* | -3.48 | -2.61 | -2.81 | -3.08 |
| SMU_RS06175 | mutanobactin A polyketide synthase |  | -2.96 | -3.00 | -3.03 | -3.03 |
| SMU_RS06185 | mutanobactin A non-ribosomal peptide synthetase |  | -3.59 | -2.62 | -3.05 | -3.08 |
| SMU_RS06190 | mutanobactin A biosynthesis thioesterase | *bacT* | -3.22 | -3.64 | -2.59 | -3.12 |
| SMU_RS06245 | class I SAM-dependent methyltransferase |  | -1.47 | -0.75 | -1.48 | -1.77 |
| SMU_RS07465 | SAM-dependent methyltransferase |  |  | 1.60 | 0.91 | 1.03 |
| SMU_RS07670 | D-alanine--poly(phosphoribitol) ligase subunit | *dltC* | 0.89 | 1.38 | 1.57 | 1.49 |
| SMU_RS07680 | D-alanine--poly(phosphoribitol) ligase subunit | *dltA* |  | -0.40 | -0.40 | -0.38 |
| SMU_RS07910 | 3-oxoacyl-[acyl-carrier-protein] reductase | *fabG* | 0.81 |  | 0.68 | 0.76 |
| SMU_RS07925 | acyl carrier protein | *acp* |  | 2.56 | 2.68 | 2.68 |

Supplementary Table 6. Differential gene expression associated with lipid metabolism

| Locus ID | Annotation | Gene | Gene expression (log_2_ FC)* | | | |
| --- | --- | --- | --- | --- | --- | --- |
|  |  |  | AA | CA | KA | DM |
| SMU_RS07880 | acetyl-CoA carboxylase carboxyl transferase subunit alpha | *accA* |  | −1.21 | −0.13 | 0.11 |
| SMU_RS07885 | acetyl-CoA carboxylase carboxyltransferase subunit beta | *accD* |  | −1.17 | −0.08 | 0.07 |
| SMU_RS07890 | acetyl-CoA carboxylase biotin carboxylase subunit | *accC* |  | −1.77 | −0.68 | −0.55 |
| SMU_RS07895 | 3-hydroxyacyl-ACP dehydratase FabZ | *fabZ* |  | −2.29 | −1.21 | −1.08 |
| SMU_RS07900 | acetyl-CoA carboxylase biotin carboxyl carrier protein | *bccP* |  | −2.65 | −1.60 | −1.45 |
| SMU_RS07905 | beta-ketoacyl-ACP synthase II | *fabF* |  | −2.41 | −1.36 | −1.24 |
| SMU_RS07915 | ACP S-malonyltransferase | *fabD* |  | −1.49 | −0.40 | −0.27 |
| SMU_RS07930 | ketoacyl-ACP synthase III | *fabH* |  | −1.84 | −0.79 | −0.73 |
| SMU_RS07940 | enoyl-CoA hydratase | *fabM* | 1.09 | −0.02 | 0.53 | 0.65 |

* Gene expression is expressed using absolute log_2_ fold change (FC) values with adjusted *p*-values less than 0.05. Red and blue correspond to up- or down-regulated gene expression levels, respectively.

Supplementary Table 7. Differential gene expression associated with cysteine and coenzyme A biosynthesis

| Locus ID | Annotation | Gene | Gene expression (log_2_ FC)* | | | |
| --- | --- | --- | --- | --- | --- | --- |
|  |  |  | AA | CA | KA | DM |
| SMU_RS05400 | O-acetylhomoserine aminocarboxypropyltransferase/cysteine synthase | *cysD* | 0.79 | 0.41 | 0.86 | 1.04 |
| SMU_RS07605 | pyridoxal phosphate-dependent aminotransferase | *metC* | 1.55 | 3.14 | 3.60 | 3.40 |
| SMU_RS07610 | cystathionine gamma-synthase | *metB* | 1.23 | 1.96 | 2.20 | 1.97 |
| SMU_RS05185 | type I pantothenate kinase | *coaA* |  | 0.88 | 1.33 | 1.40 |
| SMU_RS04950 | phosphopantothenate--cysteine ligase |  | 1.65 | 3.40 | 3.12 | 3.21 |
| SMU_RS04955 | phosphopantothenoylcysteine decarboxylase | *dfp* | 1.91 | 2.06 | 2.03 | 2.19 |
| SMU_RS02490 | pantetheine-phosphate adenylyltransferase |  | 0.99 | 1.68 | 1.86 | 1.97 |
| SMU_RS07320 | dephospho-CoA kinase |  | 1.78 | 1.79 | 2.11 | 2.07 |

* Gene expression is expressed using absolute log_2_ fold change (FC) values with adjusted *p*-values less than 0.05. Red and blue correspond to up- or down-regulated gene expression levels, respectively.

Supplementary Table 8. Differential gene expression associated with mutanobactin biosynthesis

| Locus ID | Annotation | Gene | Gene expression (log_2_ FC)* | | | |
| --- | --- | --- | --- | --- | --- | --- |
|  |  |  | AA | CA | KA | DM |
| SMU_RS06130 | mutanobactin A biosynthesis phosphopantetheinyl transferase | *mubP* |  | 1.52 |  |  |
| SMU_RS06150 | mutanobactin A system MFS transporter | *mubZ* |  | −1.05 | −1.65 | −1.82 |
| SMU_RS06155 | non-ribosomal peptide synthetase |  | −3.45 | −3.06 | −3.21 | −3.77 |
| SMU_RS06160 | non-ribosomal peptide synthetase |  | −3.79 | −2.93 | −3.27 | −3.65 |
| SMU_RS06165 | mutanobactin A non-ribosomal peptide synthetase | *mubB* | −3.66 | −3.17 | −3.23 | −3.63 |
| SMU_RS06170 | non-ribosomal peptide synthetase |  | −3.48 | −2.61 | −2.81 | −3.08 |
| SMU_RS06175 | mutanobactin A polyketide synthase | *mubH* | −2.96 | −3.00 | −3.03 | −3.03 |
| SMU_RS06180 | mutanobactin A biosynthesis transacylase | *mubG* | −3.79 | −2.54 | −2.16 | −3.55 |
| SMU_RS06185 | mutanobactin A non-ribosomal peptide synthetase | *mubE* | −3.59 | −2.62 | −3.05 | −3.08 |
| SMU_RS06190 | mutanobactin A biosynthesis thioesterase | *mubT* | −3.22 | −3.64 | −2.59 | −3.12 |
| SMU_RS06195 | mutanobactin A system ABC transporter permease subunit | *mubY* | −2.04 | −1.56 | −1.96 | −2.04 |
| SMU_RS06205 | mutanobactin A biosynthesis transcriptional regulator | *mubR* |  | 1.70 | 1.07 | 1.04 |

* Gene expression is expressed using absolute log_2_ fold change (FC) values with adjusted *p*-values less than 0.05. Red and blue correspond to up- or down-regulated gene expression levels, respectively.

1.
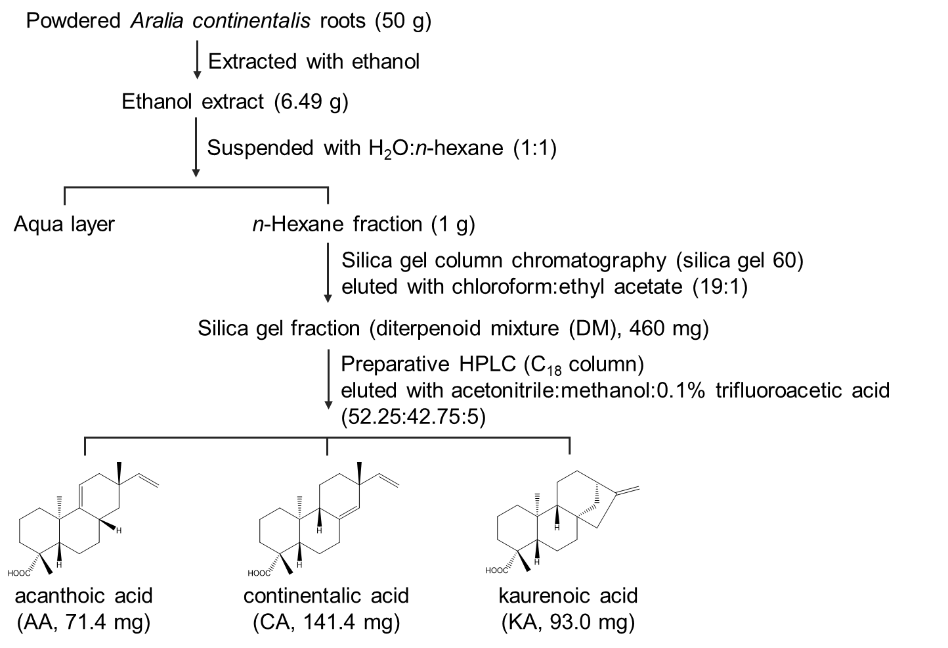

2.
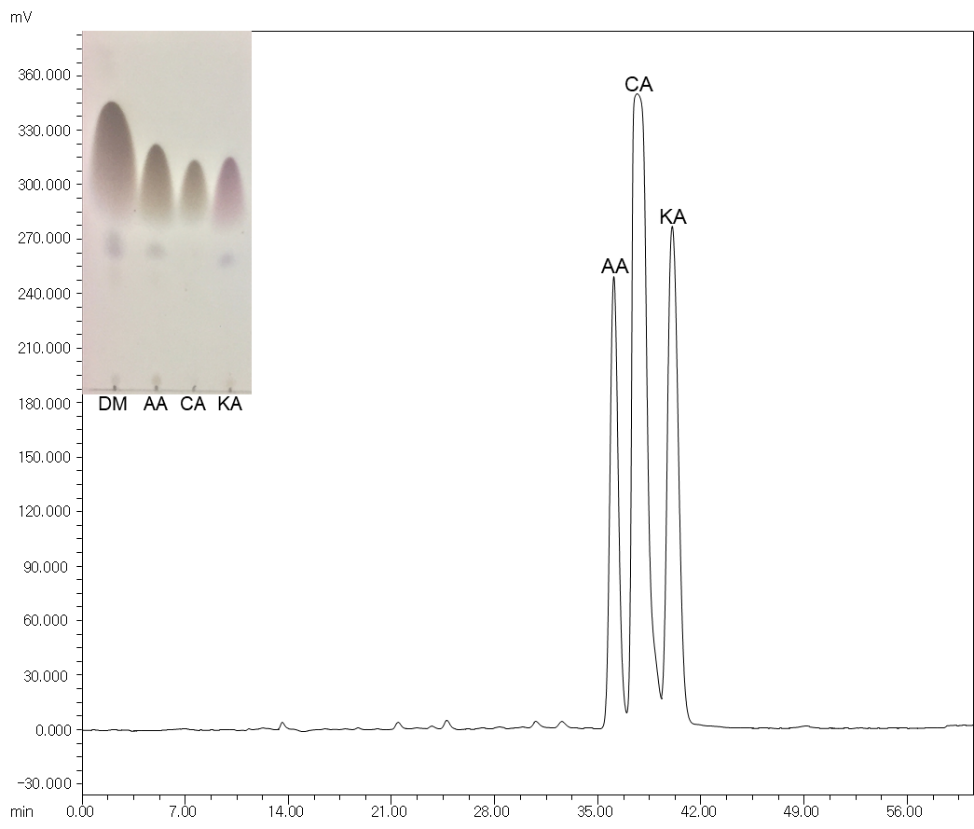

3.
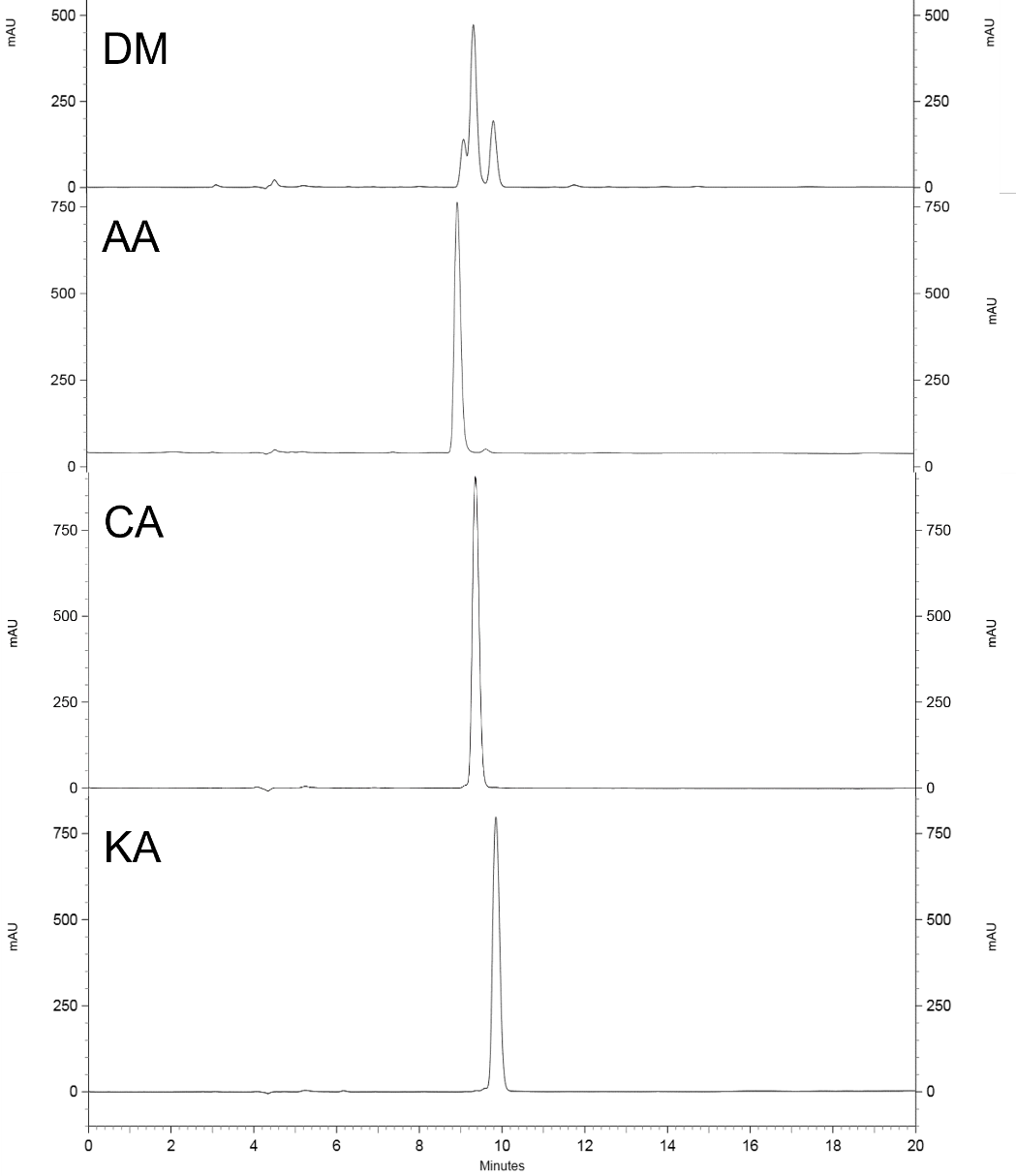

4.
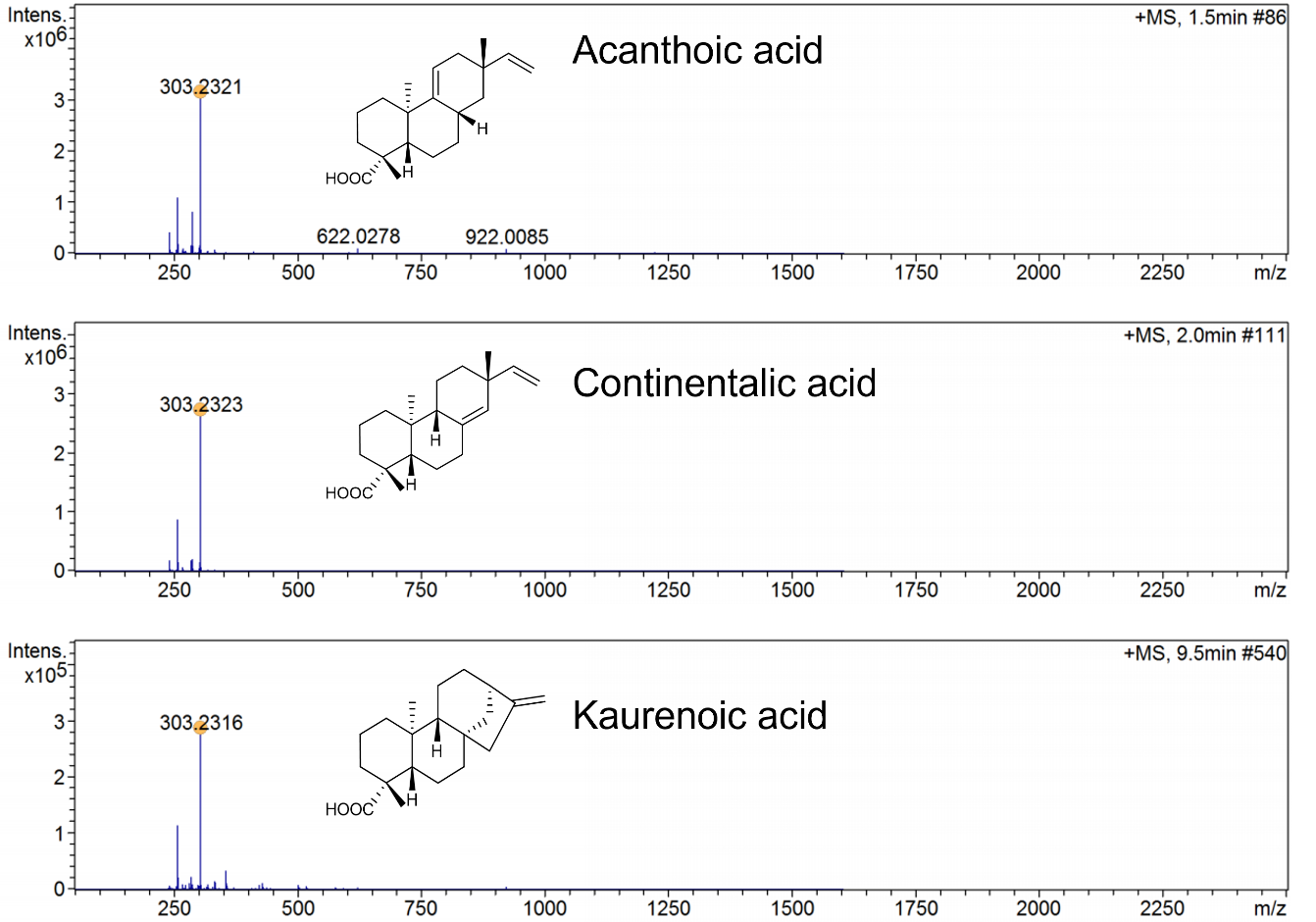

5.
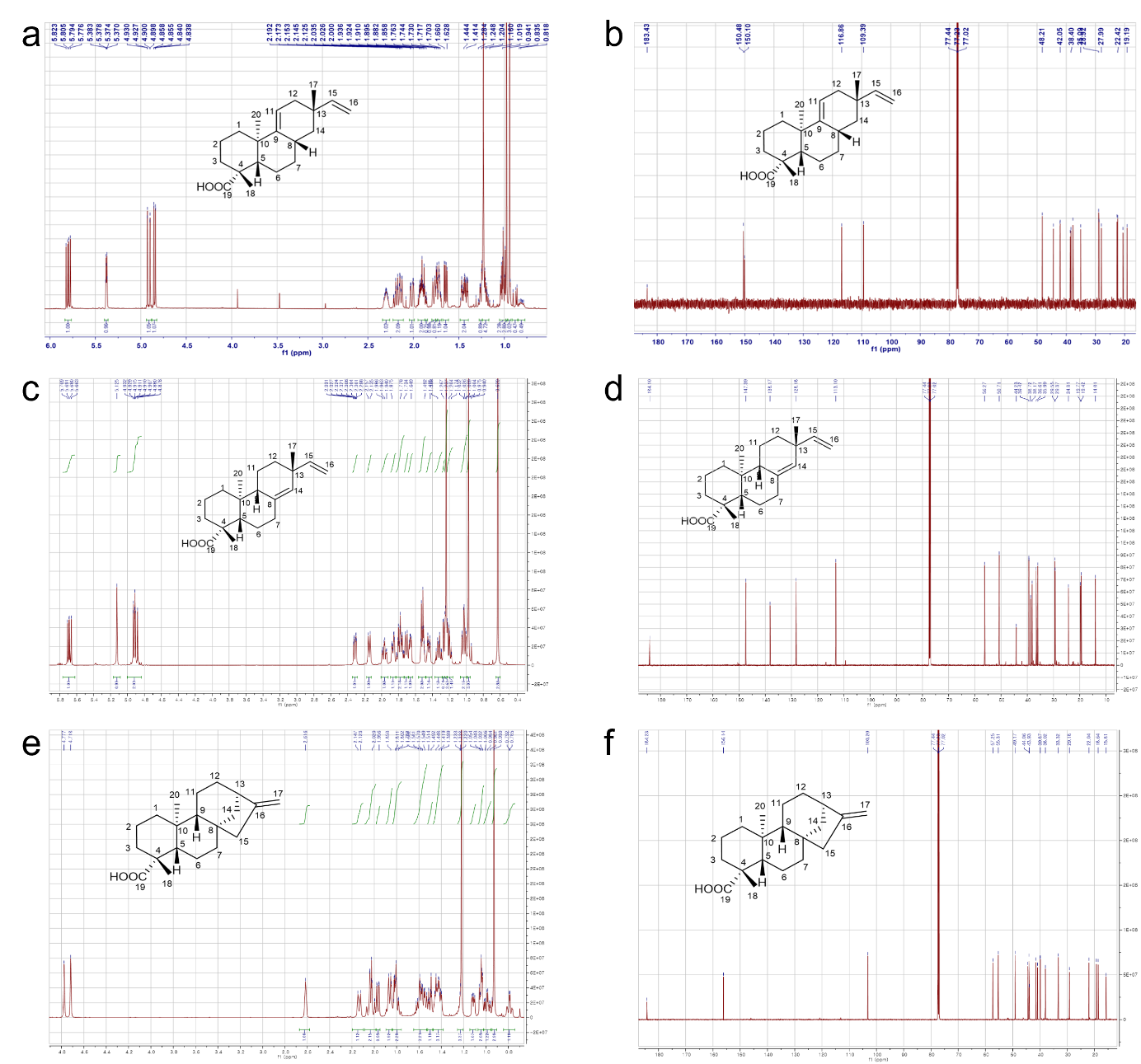


Supplementary Figure 1. Purification of diterpenoids from the roots of *Aralia continentalis*. **(A)** Schematic diagram of purification, **(B)** TLC and preparative liquid chromatography analyses of diterpenoids. TLC analysis (inlet): lane DM, diterpenoid mixture; lane AA, acanthoic acid; lane CA, continentalic acid; lane KA, kaurenoic acid. In prep LC, three diterpenoid compounds AA, CA, and KA were eluted at 36.1, 37.7, and 40.1 min, respectively, **(C)** HPLC analyses of diterpenoids, **(D)** MS analyses of diterpenoids, **(E)** NMR spectrum of three compounds AA, CA, and KA; (a) ^1^H-NMR spectrum of acanthoic acid (600 MHz, CDCl_3_), (b) ^13^C-NMR spectrum of acanthoic acid (150 MHz, CDCl_3_), (c) ^1^H-NMR spectrum of continentalic acid (600 MHz, CDCl_3_), (d) ^13^C-NMR spectrum of continentalic acid (150 MHz, CDCl_3_), (e) ^1^H-NMR spectrum of kaurenoic acid (600 MHz, CDCl_3_), (f) ^13^C-NMR spectrum of kaurenoic acid (150 MHz, CDCl_3_).


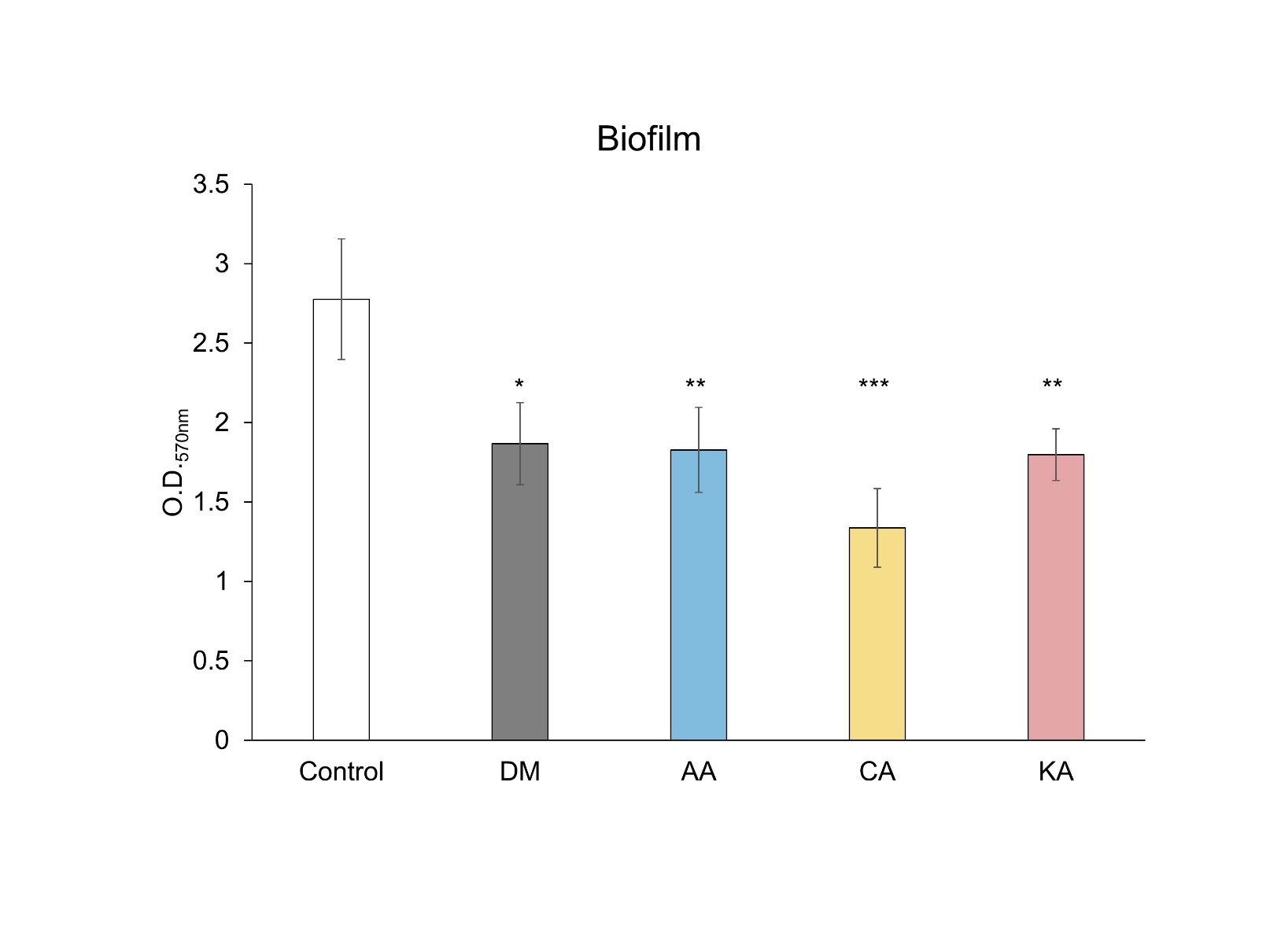


Supplementary Figure 2. The anti-biofilm activities of diterpenoids against *S. mutans* were determined by crystal violet staining. Significant differences were observed when compared with the control: * p < 0.05, ** p < 0.005, *** p < 0.001.

1.
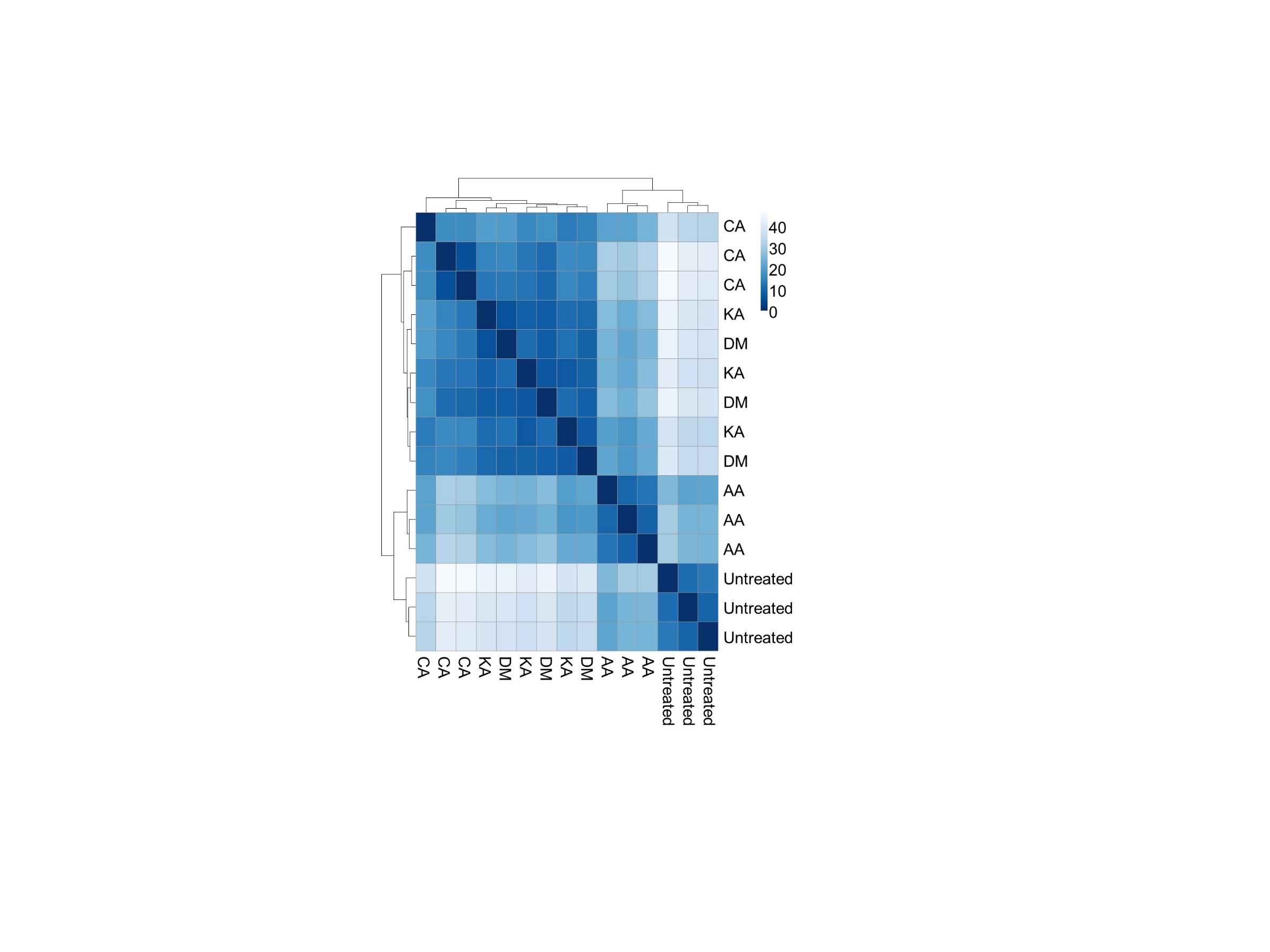

2.
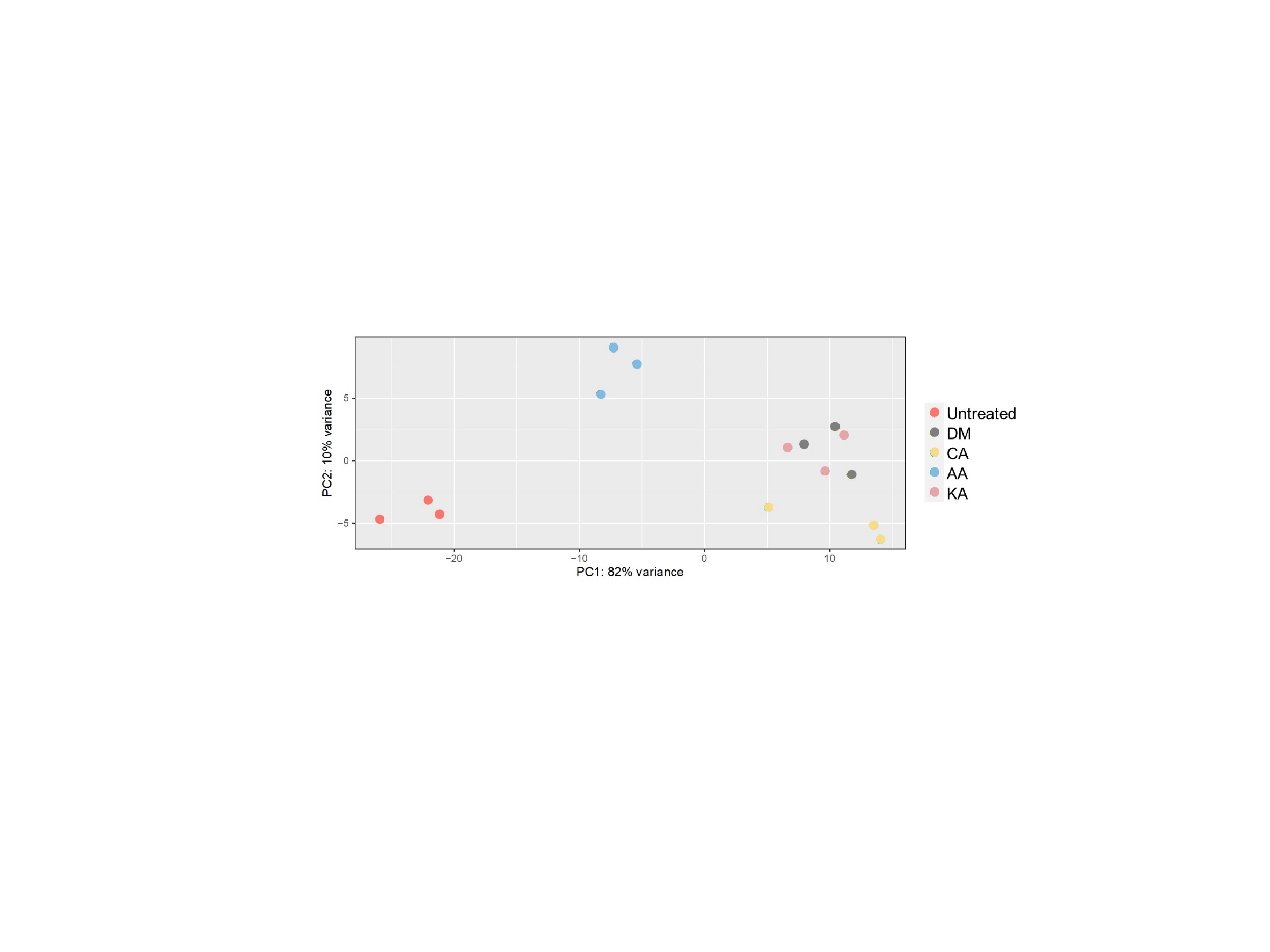

3.
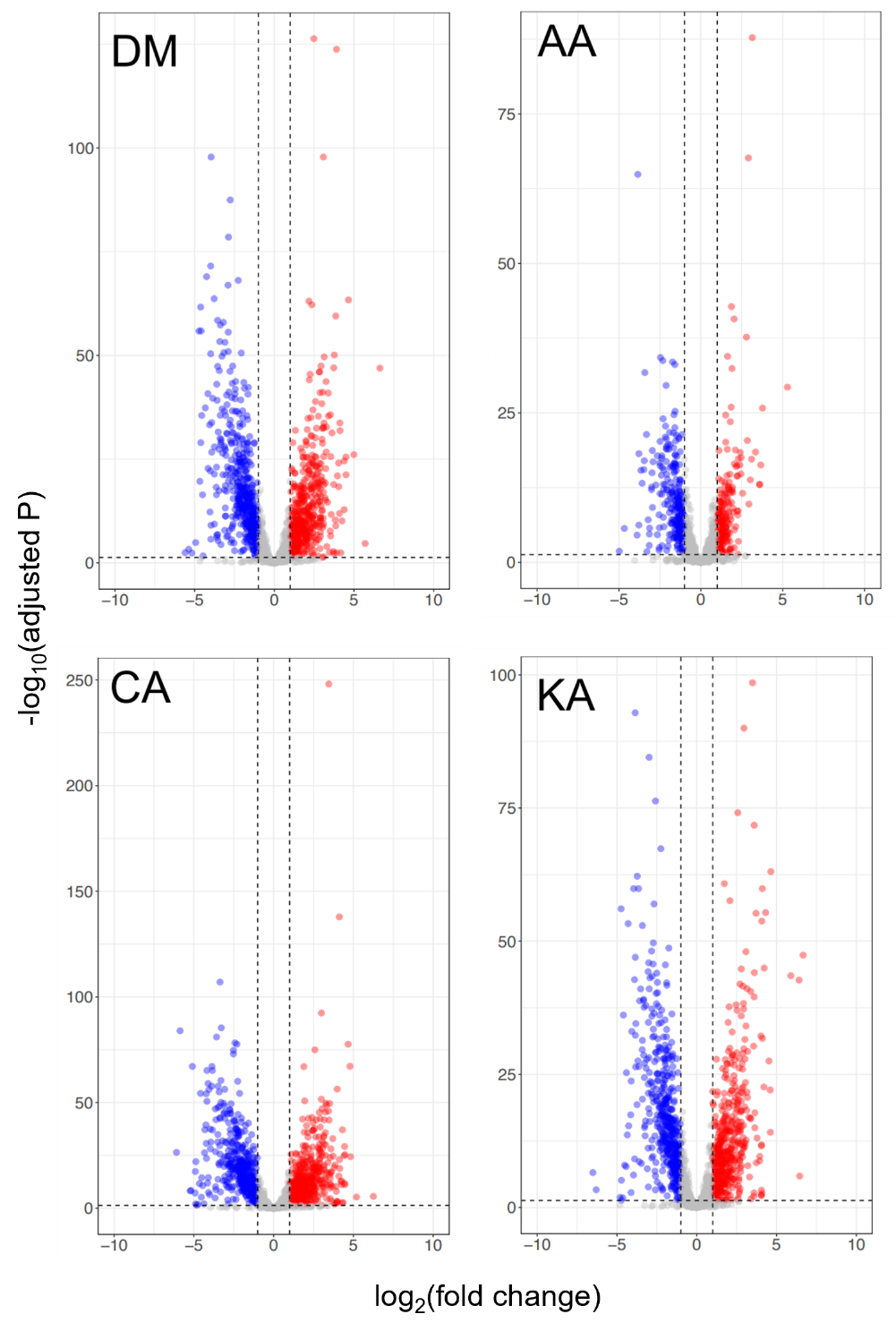


Supplementary Figure 3. Cluster analysis of RNA-seq data. (A) Heatmap of the distances between samples by Euclidean method, (B) principal component analysis (PCA) plot of the overall gene expression and (C) Volcano plot for DEGs. DEGs with >1.0-fold change and *p* < 0.05 were clustered across all the samples.

1.
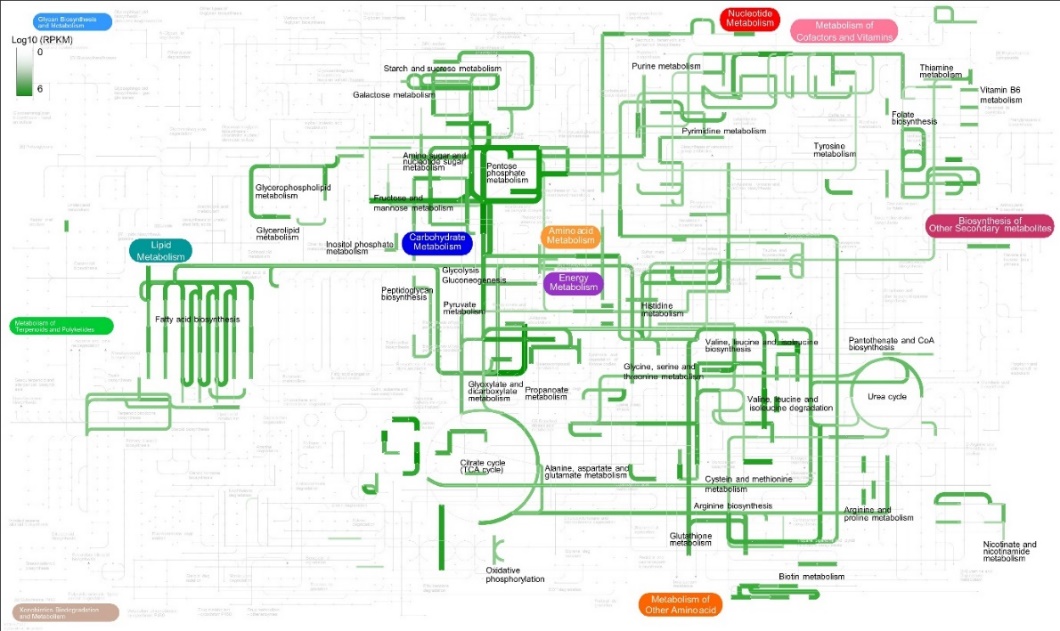

2.
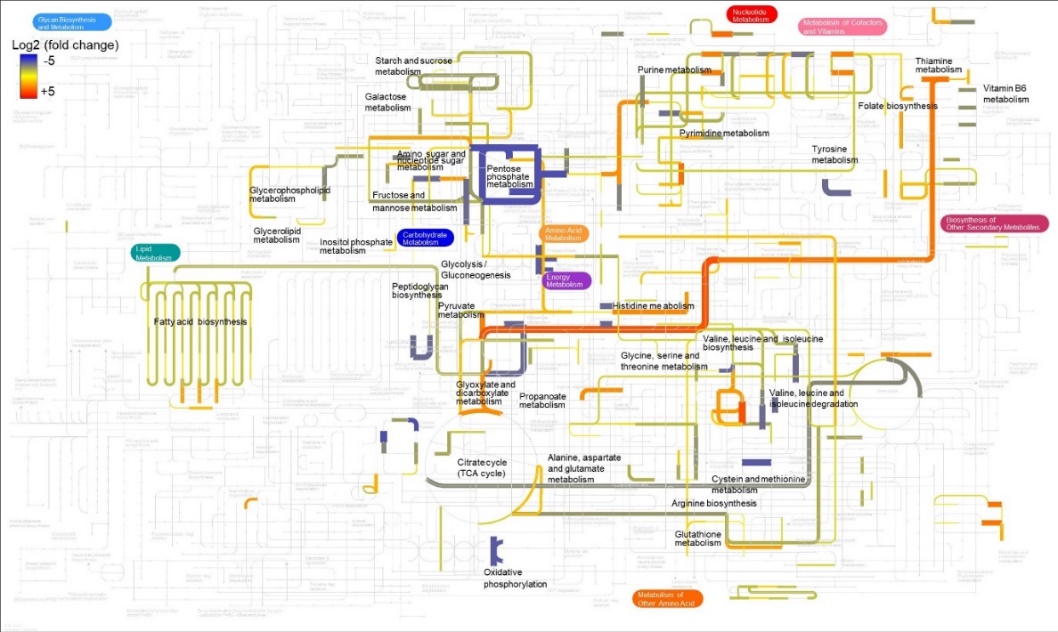

3.
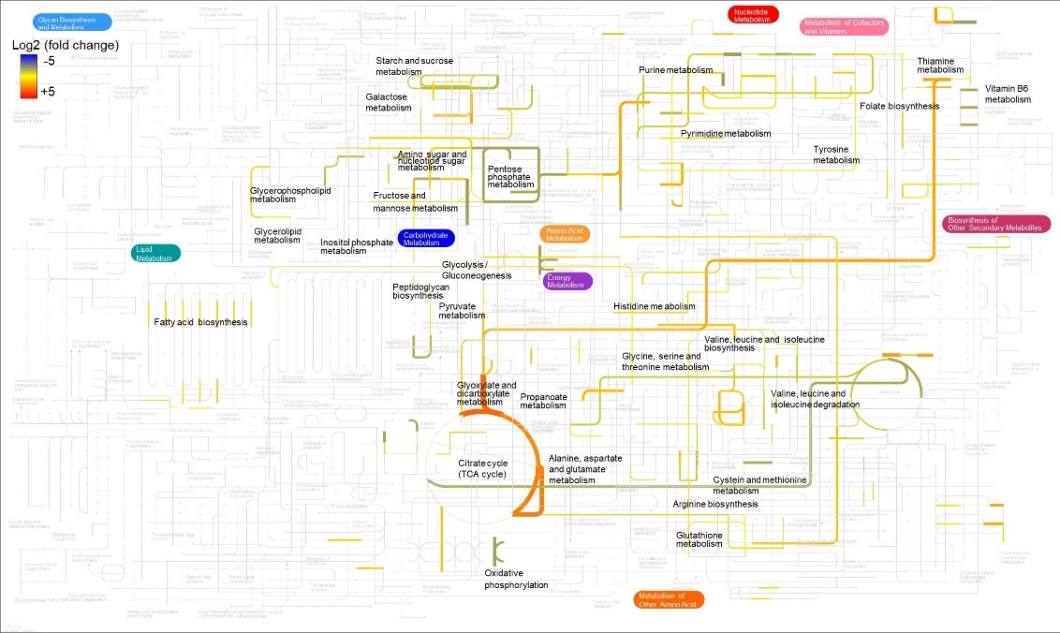

4.
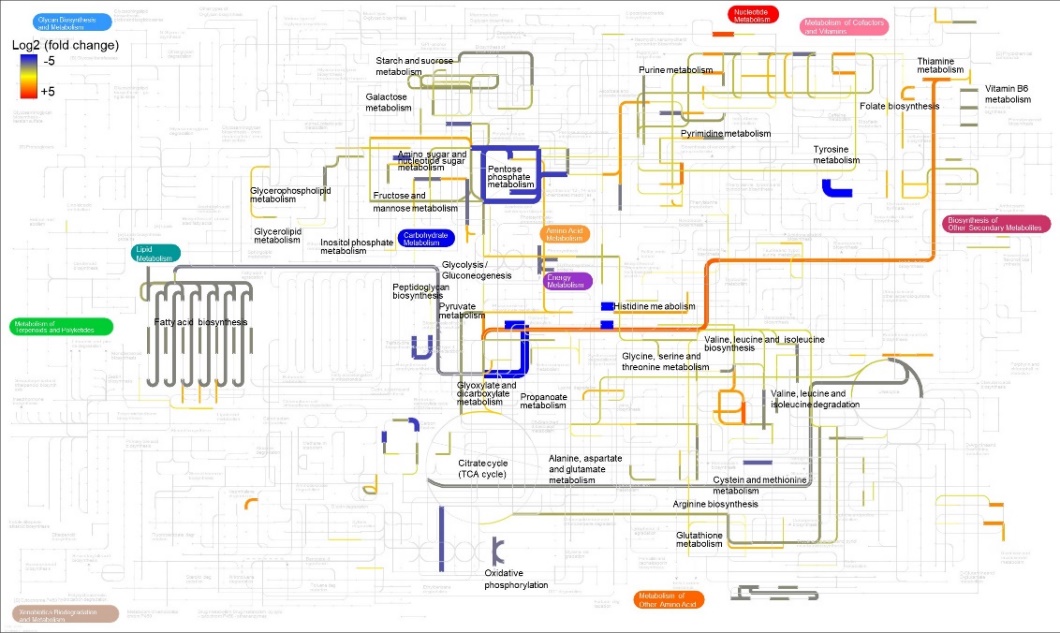

5.
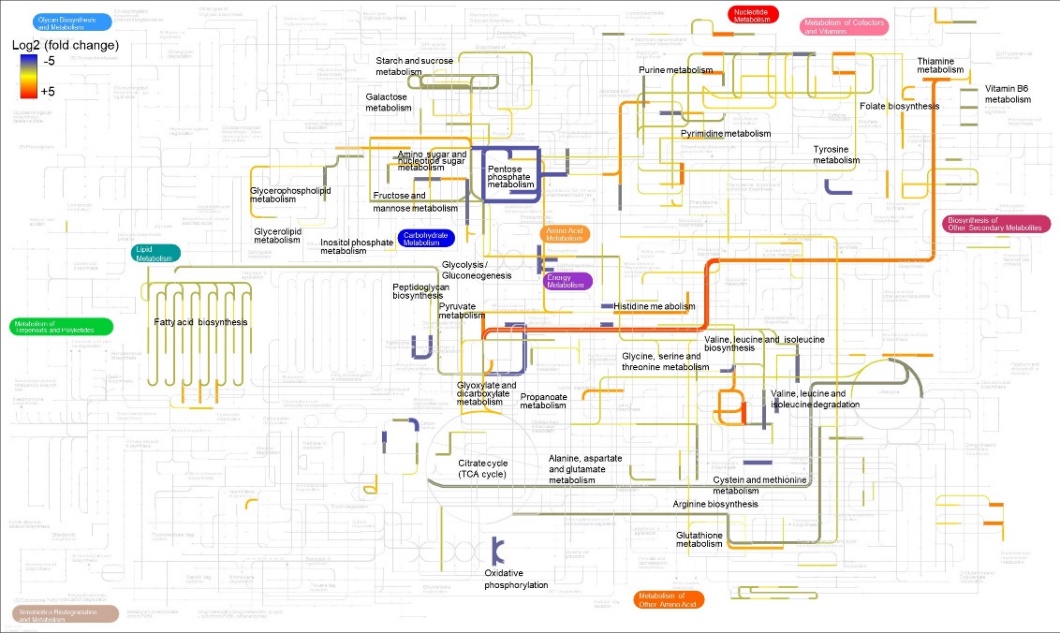


Supplementary Figure 4. Metabolic pathways of *S. mutans* UA159. The pathways were generated using the iPath (ver. 3.0) module and are based on the KEGG Orthology numbers of genes identified in the genome of *S. mutans* UA159. (A) Transcriptional expression of the metabolic pathway of *S. mutans* UA159 without the addition of diterpenoids. Transcriptional expression of the metabolic pathway of *S. mutans* UA159 treated with (B) DM, (C) AA, (D) CA, and (E) KA at a concentration of 4 μg/mL for 1 h.
